# 2018 Survey of factors associated with antimicrobial drug use and stewardship practices in adult cows on conventional California dairies: immediate post-Senate Bill 27 impact

**DOI:** 10.7717/peerj.11596

**Published:** 2021-07-13

**Authors:** Pius S. Ekong, Essam M. Abdelfattah, Emmanuel Okello, Deniece R. Williams, Terry W. Lehenbauer, Betsy M. Karle, Joan D. Rowe, Sharif S. Aly

**Affiliations:** 1 Veterinary Medicine Teaching and Research Center, University of California, Davis, Tulare, CA, United States; 2 Department of Epidemiology, National Veterinary Research Institute, Vom, Plateau, Nigeria; 3 Department of Animal Hygiene and Veterinary Management, Faculty of Veterinary Medicine, Benha University, Qalubiya Governorate, Egypt; 4 School of Veterinary Medicine, Department of Population Health and Reproduction, University of California, Davis, California, United States; 5 Cooperative Extension, Division of Agriculture and Natural Resources, University of California, Orland, California, United States

**Keywords:** California dairy industry, Antimicrobial drug use, Antimicrobial stewardship, Judicious use of antibiotics, Risk factors, Logistic regression, Machine learning, Decision tree, Random forest, Gradient boosting

## Abstract

**Background:**

Antimicrobial drugs (AMD) are critical for the treatment, control, and prevention of diseases in humans and food-animals. Good AMD stewardship practices and judicious use of AMD are beneficial to the preservation of animal and human health from antimicrobial resistance threat. This study reports on changes in AMD use and stewardship practices on California (CA) dairies, following the implementation of CA Senate Bill 27 (SB 27; codified as Food and Agricultural Code, FAC 14400–14408; here onward referred to as SB 27), by modeling the associations between management practices on CA conventional dairies and seven outcome variables relating to AMD use and stewardship practices following SB 27.

**Methods:**

A survey questionnaire was mailed to 1,282 grade A licensed dairies in CA in spring of 2018. Responses from 132 conventional dairies from 16 counties were included for analyses. Multivariate logistic regression models were specified to explore the associations between survey factors and six outcome variables: producers’ familiarity with the Food and Drug Administration’s (FDA), Silver Spring, WA, USA medically important antimicrobial drugs (MIAD) term; change in over-the-counter (OTC) AMD use; initiation or increased use of alternatives to AMD; changes to prevent disease outbreaks; changes in AMD costs; and better animal health post SB 27. We employed machine learning classification models to determine which of the survey factors were the most important predictors of good-excellent AMD stewardship practices of CA conventional dairy producers.

**Results:**

Having a valid veterinary-client-patient-relationship, involving a veterinarian in training employees on treatment protocols and decisions on AMDs used to treat sick cows, tracking milk and/or meat withdrawal intervals for treated cows, and participating in dairy quality assurance programs were positively associated with producers’ familiarity with MIADs. Use or increased use of alternatives to AMDs since 2018 was associated with decreased use of AMDs that were previously available OTC prior to SB 27. Important variables associated with good-excellent AMD stewardship knowledge by CA conventional dairy producers included having written or computerized animal health protocols, keeping a drug inventory log, awareness that use of MIADs required a prescription following implementation of SB 27, involving a veterinarian in AMD treatment duration determination, and using selective dry cow treatment.

**Conclusions:**

Our study identified management factors associated with reported AMD use and antimicrobial stewardship practices on conventional dairies in CA within a year from implementation of SB 27. Producers will benefit from extension outreach efforts that incorporate the findings of this survey by further highlighting the significance of these management practices and encouraging those that are associated with judicious AMD use and stewardship practices on CA conventional dairies.

## Introduction

Antimicrobial drugs (AMDs) are natural or synthetic products that destroy or inhibit the growth of microbes or prevent or counteract their pathogenic action. They are critical for the treatment, control, and prevention of diseases in humans and animals (Jayarao, Almeida & Oliver, 2019; Oliver, Murinda & Jayarao, 2011). According to the US Food and Drug Administration (FDA), approximately 6.1 million kgs of medically important AMD were sold or distributed for use in food-producing animals in USA in 2019, representing a 3% increase from 2018 (FDA (U.S Food and Drug Administration), 2020). Inappropriate or unregulated use of AMD has been linked with increased risk of antimicrobial resistance (AMR) affecting both human and animal health (CDC (U.S. Centers for Disease Control and Prevention), 2019; WHO (World Health Organization), 2015). AMR is an emerging public health threat imposing significant health and economic burdens to the global population (Davies & Wales, 2019; Stelling et al., 2020). Implementation of effective AMD stewardship practices and judicious use of AMD are critical to the reduction of AMR threat to animal and public health (Tang et al., 2017).

In many countries, substantial efforts have been made to reduce the overall use of AMD in food-producing animals by encouraging good AMD stewardship practices that included bans of AMD in the feed of food-producing animals, benchmarking AMD use at the farm level, and antibiotic susceptibility testing (EMA & EFSA, 2017). On January 1, 2018, California (CA) implemented Senate Bill 27 (SB 27; codified as Food and Agricultural Code, FAC 14400–14408; here onwards referred to as SB 27), becoming the first state in the US to require a veterinary prescription for all medically important AMDs (MIADs) used for therapy, in addition to regulations already passed under the Food and Drug Administration’s (FDA), Silver Spring, WA, USA Veterinary Feed Directive (VFD) requiring veterinary oversight for MIADs administered through feed *CA* (CA (California) Senate, 2015; CDFA (California Department of Food and Agriculture), 2019a; FAC (Food and Agricultural Code), 2015). The SB 27 mandated the development of antimicrobial stewardship guidelines and resources that support the collection of information on livestock management practices, AMD sales, AMD use, and AMR in order to provide relevant information to producers and other stakeholders.

For several years, a broad array of stakeholders, including medical and veterinary AMR specialists, researchers, animal agriculture representatives, environmental and consumer advocates, and leaders in State government, participated in the development of comprehensive legislation intended to prolong the effectiveness of antibiotics, culminating in the passage of Senate Bill 27 in 2015. The shared goal was to improve the ability to provide for public health, animal health, and environmental health into the future, while not overregulating the practice of veterinary medicine. During the process, antimicrobial stewardship models used in human medicine, veterinary models used in several countries, and the United States’ *National Action Plan for Combatting Antimicrobial Resistant Bacteria* were reviewed; and, first-person accounts of their strengths and weaknesses–as identified by those responsible for implementation-contributed to the California effort. As the legislation started to take shape, the leadership of the sponsoring State Senator, the Governor, and the State Veterinarian were instrumental in bringing stakeholders, often with diverse opinions, together to develop optimal solutions. While several side agendas arose, the group was able to continually refocus on the shared goal of preserving antibiotics while faced with the realization of the complexities involved. This understanding led to a comprehensive legislative approach and the funding needed to arm veterinarians and livestock owners with science-based recommendations, data-gathering that looks beyond just quantity to clinical impact over time, and a proactive move to limit AMD use and require veterinary oversight for all use of MIADs, as is required in human medicine.

Funded by the CA Department of Food and Agriculture (CDFA), Sacramento, CA, USA the supervising branch for the Antimicrobial Use and Stewardship Program established by the SB 27, we conducted a survey of AMD use on adult cows and stewardship practices on CA dairies immediately after the implementation of the SB 27. This statewide survey was designed to collect information about reported AMD use in adult cows, AMD stewardship practices, and the immediate impact of the SB 27 and VFD laws on CA dairies. A detailed description of the survey tool and descriptive statistics on the producer responses to questions related to AMD use and stewardship practices are presented elsewhere (Ekong et al., 2021).

The aim of this study was to determine the factors associated with AMD use and stewardship practices in adult cows on conventional CA dairies and reported changes since the implementation of SB 27. Specifically, our study objectives were to model the association between different management practices on conventional dairies and six outcome variables, which are: (1) producers’ familiarity with FDA’s MIAD term, (2) decreased use of AMDs previously available over-the-counter (OTC), and (3) initiation or increased use of alternatives to AMD post SB 27. Additional modeled outcomes included (4) whether producer’s made changes to prevent disease outbreaks, (5) reported changes in AMD costs, and (6) reported better animal health on their dairies post SB 27. Finally, (7), we employed machine learning algorithms to determine which of the survey factors were the most important predictors of good-excellent antimicrobial stewardship practices among CA conventional dairy producers.

## Methodology

### Schematic overview of study

**Figure 1:**
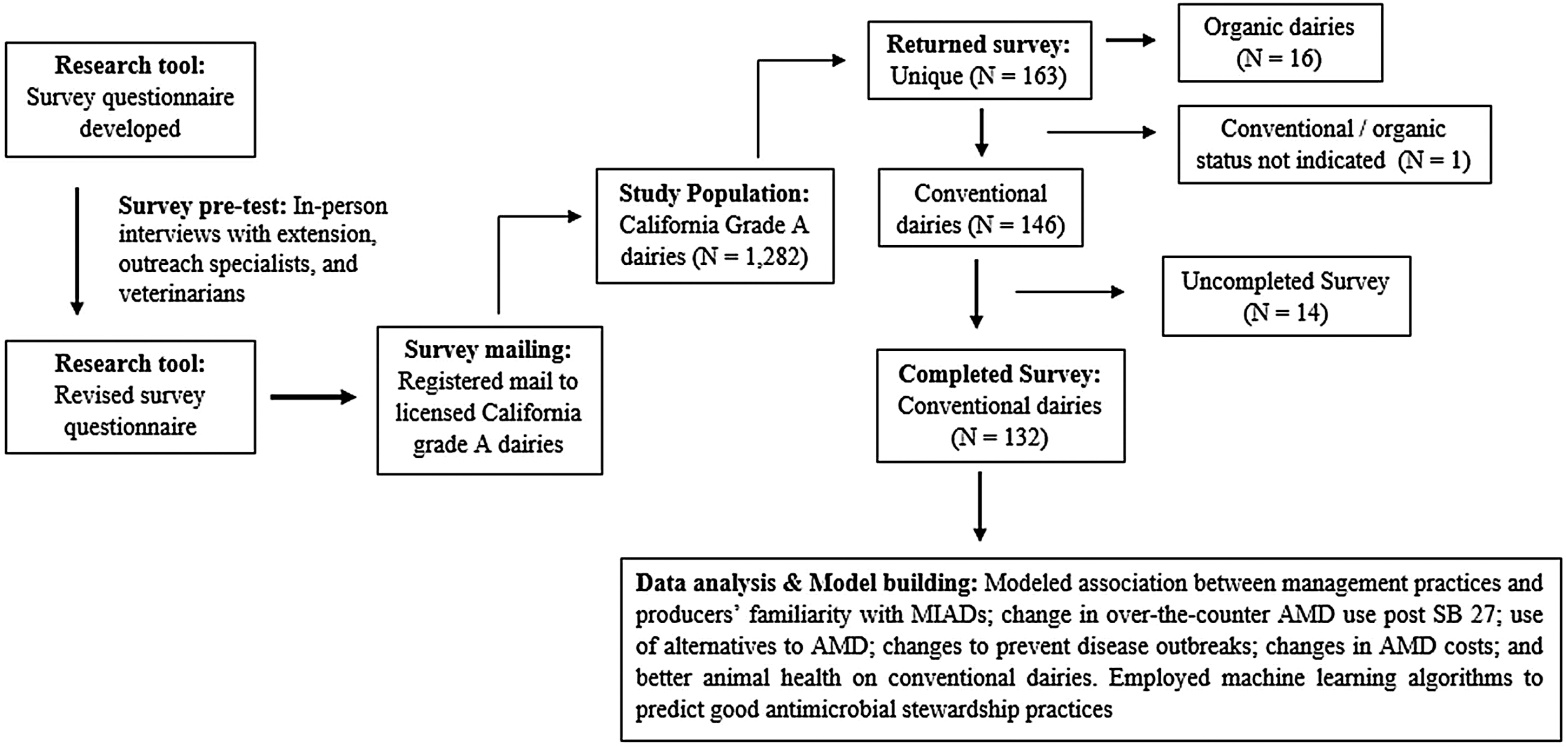
Schematic overview of development and activity for a survey of Medically Important Antimicrobial Drug (MIAD) use and stewardship practices in adult cows on California dairies after Senate Bill (SB) 27 regulations.

The survey development, sections and activity are described in detail in Ekong et al. (2021) and summarized in Fig. 1 here for the conventional dairies only.

### Development and administration of research instrument

Briefly, a survey questionnaire was designed by researchers including epidemiologist, veterinary clinician, extension specialist, and postdoctoral scholars to collect information about AMD use in adult cows on CA dairies. The questionnaire was pre-tested using in-person interviews with extension and outreach specialists and veterinarians. A survey packet containing a copy of the final questionnaire, a postage-paid addressed business reply envelope, and an information cover letter was registered mailed to 1,282 licensed grade A dairies in CA. The cover letter included explanation to producers that the term antimicrobial included antibiotics, drugs that are naturally produced by other microorganisms and that can kill or inhibit the growth of other microorganisms, and also synthetic chemicals such as sulfonamides. Producers were instructed that, for the purpose of this survey, questions referred to all antimicrobial drugs as antibiotics regardless of their origin or other technical distinctions. To improve survey response rate, dairies that did not respond to the first mailing of the questionnaire received a second and a third mailing of the questionnaire every two months after the initial questionnaire package. Mailings of questionnaire occurred between April and September 2018. Finally, completed and returned questionnaires were scanned and data entered in Microsoft Excel. For this study, only data from conventional dairies were analyzed, data from organic dairies were excluded.

### Questionnaire content and survey process

The questions on the survey were categorized under three sections, namely: (1) herd information; (2) dairy cow health management and AMD use; and (3) practices and perspectives. The first section gathered baseline information about the responding dairy, including: the respondent’s role, the county where the dairy was located, the breeds and number of milking cows, the annual rolling herd average for milk production, the previous month’s average bulk tank somatic cell count, and if the dairy was a USDA certified organic milk producer. The questions on the second section addressed farms’ dry-off protocols, vaccination, disease prevention, diagnosis and management practices, sources of information on AMDs, how decisions on AMDs stocked or used are made, and whether producers had written or computerized animal health protocols or used a drug inventory log on their dairies. Other questions included AMD treatment information and withdrawal tracking, and if the dairy had a working relationship with a veterinarian or veterinary practice. The final section addressed questions relating to the respondent’s participation in animal welfare audit programs and/or dairy quality assurance programs, familiarity with the MIADs, changes made regarding AMDs previously available OTC, changes in the dairy’s AMD drug costs, usage of alternatives to AMDs, changes in management to prevent disease, and the herd’s health status that may have occurred in the periods before and after January 1, 2018 (pre-and post-SB 27). Finally, survey responses from 132 conventional dairy producers were used in the current study after excluding responses from organic producers.

### Statistical methods

#### Descriptive statistics

For analytical purposes, the locations of respondent dairies were classified into three regions: Northern California (NCA), Northern San Joaquin Valley (NSJV), and Greater Southern California (GSCA) (Love et al., 2016). Proportions and their standard errors were computed for categorical and ordinal variables. Means and standard errors were computed for continuous variables. Confidence intervals for proportions were calculated using the normal distribution approximation method (Daniel, 2005). Descriptive analyses were performed using Stata 15 (StataCorp, College Station, TX, USA).

### Multivariable logistic regression model

Six survey questions were selected and analyzed as outcome (dependent) variables. They included the reported (1) familiarity with FDA’s “medically important antimicrobial drugs” term (familiarity with MIADs); (2) changes made since January 2018 regarding use of injectable, bolus and/or intramammary dosage forms of AMD that were previously available OTC as compared to their use in 2017 (changes made regarding OTC AMD); (3) initiation or increased use of alternatives to AMDs since January 2018 (use of alternatives to AMDs); (4) changes in management to prevent disease outbreak or spread since January 2018 (made changes to prevent disease); (5) description of farm’s AMD drug costs since January 2018 compared to 2017 and earlier (farm AMD costs); and (6) description of farm’s animal health conditions since January 2018 compared to 2017 and earlier (herd’s health status).

Familiarity with MIADs, as reported by Ekong et al., 2021, was identified if the survey respondent recognized the FDA classification of medically important antimicrobial drugs (MIADs) and/or that MIADs are available for livestock only via prescription or veterinary feed directive pursuant to a veterinary-client-patient relationship (VCPR) with a licensed veterinarian. Familiarity with MIADs was dichotomized into “familiar” and “not familiar”. The second outcome modeled was changes made regarding OTC AMD and was classified as “less” or “more” AMDs used since January 2018 compared to 2017. The third outcome was initiation or increased use of alternatives to AMDs in 2018 compared to 2017 which was dichotomized as “yes” (use alternatives) or “no” (do not use alternatives). The fourth outcome regarding changes made to prevent disease, such as improvements in vaccination programs to prevent disease, quarantine of purchased or returning animals from offsite locations, improved biosecurity, and pre-purchase testing of animals before adding to the herd, was dichotomized as “yes” (made changes) and “no” (no changes). Both the fifth and sixth outcomes, changes in farm AMD drug costs and animal health conditions in 2018 were dichotomized as “decreased” or “increased/no change”, and “better” or “worse/no change”, respectively. Mixed effect logistic regression models with a random effect for dairy were fitted to each outcome. Univariate mixed effect models were first fitted for each of the outcomes using each of the survey variables described in Ekong et al., 2021, including herd demographics, dairy cow health management and AMD use, and dairy practices and perspectives. Manual forward multivariate model building was performed, educated by the association between survey variables and outcomes while assessing for potential confounding by breed, milking herd size, annual rolling herd average (RHA) for milk production, and region, using the method of change in estimates (Aly et al., 2010). All biologically meaningful interaction terms were explored using significance testing. Final model selection and fit were assessed using information theory by monitoring estimates of the Akaike information criterion (AIC).

### Machine learning classification models

Three machine learning (ML) predictive models, decision tree (DT), random forest (RF), and gradient boosting (GB) algorithms (Amrine et al., 2019; Breiman, 2001; Fei et al., 2017; Friedman, 2002; Strobl, Malley & Tutz, 2009), were used to identify the features of dairies and producers who considered AMD stewardship practices as important, based on a single survey question. The question requested respondents to classify these five AMD stewardship practices as very important, somewhat important or not important: (1) Administration of appropriate AMD dose, route and duration; (2) Good record keeping on treatment and treatment dates; (3) Having a current VCPR; (4) Observing withdrawal periods and drug residue avoidance; and (5) Using alternatives to AMD. Producers were given a score of one to five, based on the number of the AMD use stewardship questions that they scored as very or somewhat important. A score of five was ranked “excellent”, four as “good”, three as “moderate”, and two or one as “limited” knowledge of AMD use stewardship. Respondent’s dairies were classified as having “good-excellent” AMD use stewardship knowledge based on a score of ≥4 or as having “limited-moderate” AMD use stewardship knowledge based on a score of ≤3. Having “good-excellent” versus “limited-moderate” AMD use stewardship knowledge was the outcome of the ML models. Each ML model was offered a set of 21 survey factors that contributed to the majority of the variability in responses, as identified using a Multiple Factor Analysis (MFA) reported by Ekong et al., 2021. The survey variables included continuous (herd size; RHA; and Somatic Cell Count, (SCC)) and categorical variables. The categorical variables included region, breed, and management practices (selective dry cow treatment, vaccination against mastitis). Other variables offered to the model related to AMD use and tracking, such as sources of information, tracking of AMD usage by keeping computer records alone or in combination with other methods, tracking of AMD withdrawal intervals by keeping computer records alone or in combination with other methods, treatment of cows with AMD previously available OTC before January 2018, and reduced use of AMD previously available OTC since the start of 2018. Other methods used to track AMD use and withdrawal intervals included paper records, white- or chalkboard, markings on the animal, or from memory. Finally, survey factors associated with AMD stewardship, such as written or computerized animal health protocols, drug inventory logs, participation in animal welfare audit programs, familiarity with MIADs, and awareness that MIADs require a prescription since January 2018, were also explored. The dataset was partitioned into training and testing sets using a random split ratio 70:30 (training:test). The breakdown of the training dataset by region was: NCA 14.2%, NSJV 40.7%, and GSCA 45.1%, while the testing dataset was NCA 13.9%, NSJV 33.3%, and GSCA 52.8%. Each model was trained with the training dataset and evaluated by assessing their predictive performance on the testing dataset using Salford Predictive Modeler 8.0 (SPM) software (https://www.salford-systems.com/products/spm/userguide). The DT model builds classification trees by selecting the most important predictors from a large number of variables to explain the outcome (Zhang, 2012). For the DT algorithm, we used a 10-fold cross validation method for testing, Gini as the optimization method, and minimum cost tree as the choice for the best tree. The RF model builds several individual classification trees using a random subsample of the data and then selects the most popular class, i.e., mean prediction of the individual trees (Breiman, 2001). For the RF, we used the out-of-bag testing method, with 500 classification trees, three predictors considered for each node, and balanced sample weights. The GB is an optimization algorithm that combined the efforts of multiple classification trees using a random subsample of the data to produce a strong class with reduced prediction variance (Chen & Guestrin, 2016; Rikin et al., 2015; Zhang et al., 2019). For the GB model, we used a 10-fold cross validation, tree size of 500, balanced sample weights, and best model chosen by cross entropy. Model performance diagnostics included measures of model accuracy, sensitivity, specificity, precision, F1 score, Matthew’s correlation coefficient (MCC), and Area Under Curve (AUC) estimated from receiver operator characteristic (ROC) curve analyses which were all assessed and compared across the three machine learning algorithm (DT, RF, GB) models (Maier et al., 2019a). The importance of predictors was determined by their relevance ranks, the latter a function of the aforementioned performance diagnostics.

## Results and discussions

The herd characteristics of the dairy producers included in the analyses are summarized in Table 1. The majority of the responses were from GSCA (50.8%) and NSJV (43.2%) compared to NCA (6%), with the predominant breed being Holstein (69.5%). For the purposes of analysis, milking herd size was categorized as <1,305, the state mean; 1,305–3,500; and >3,500 milking cows, while annual rolling herd average for milk production was categorized as <10,660 kg/cow (23,500 lbs/cow), the state mean; and ≥10,660 kg/cow (CDFA (California Department of Food and Agriculture), 2018a). Approximately half (51.5%) of the dairies had a milking herd size that was <1,305 milking cows, a third (34.5%) had an annual rolling herd average milk production that was <10,660 kg/cow and 69.5% reported bulk tank somatic cell counts <200,000 cells/ml.

**Table 1:** Summary of herd information from 132 responses to a survey questionnaire on antimicrobial drug use in adult cows on conventional California dairies.

Question	*n*	Estimate (%)	SE	95% Confidence limits	
				Lower	Upper
**Region**					
GSCA^a^	67	50.8	4.3	42.2	59.2
NCA^b^ + NSJV^c^	65	49.2	4.3	40.7	57.8
**Breed**					
Holstein	91	69.5	4.0	60.9	76.8
Jersey	5	3.8	1.6	1.5	8.9
Mixed/Other	35	26.7	3.8	19.7	35.0
**Herd size**					
<1,305	68	51.5	4.3	42.9	59.9
1,305–3,500	56	42.4	4.3	34.2	51.0
>3,500	8	6.1	2.1	3.0	11.7
**Rolling herd average (kg/cow)**					
<10,660	41	34.5	4.3	26.4	43.5
≥10,660	78	65.5	4.3	56.5	73.6
**Bulk tank somatic cell count (cells/mL)**					
<100,000	15	11.5	2.7	6.9	18.2
100,000–199,999	76	58.0	4.3	49.3	66.2
≥200,000	40	30.5	4.0	23.1	39.0

**Notes:**

a GSCA = Greater southern California.

b NCA = Northern California (8 respondent dairies).

c NSJV = Northern San Joaquin Valley (57 respondent dairies).

A rolling herd average of 10,660 kg/cow is equivalent to 23,500 lbs/cow.

### Multivariate logistic regression model

**Figure 2:**
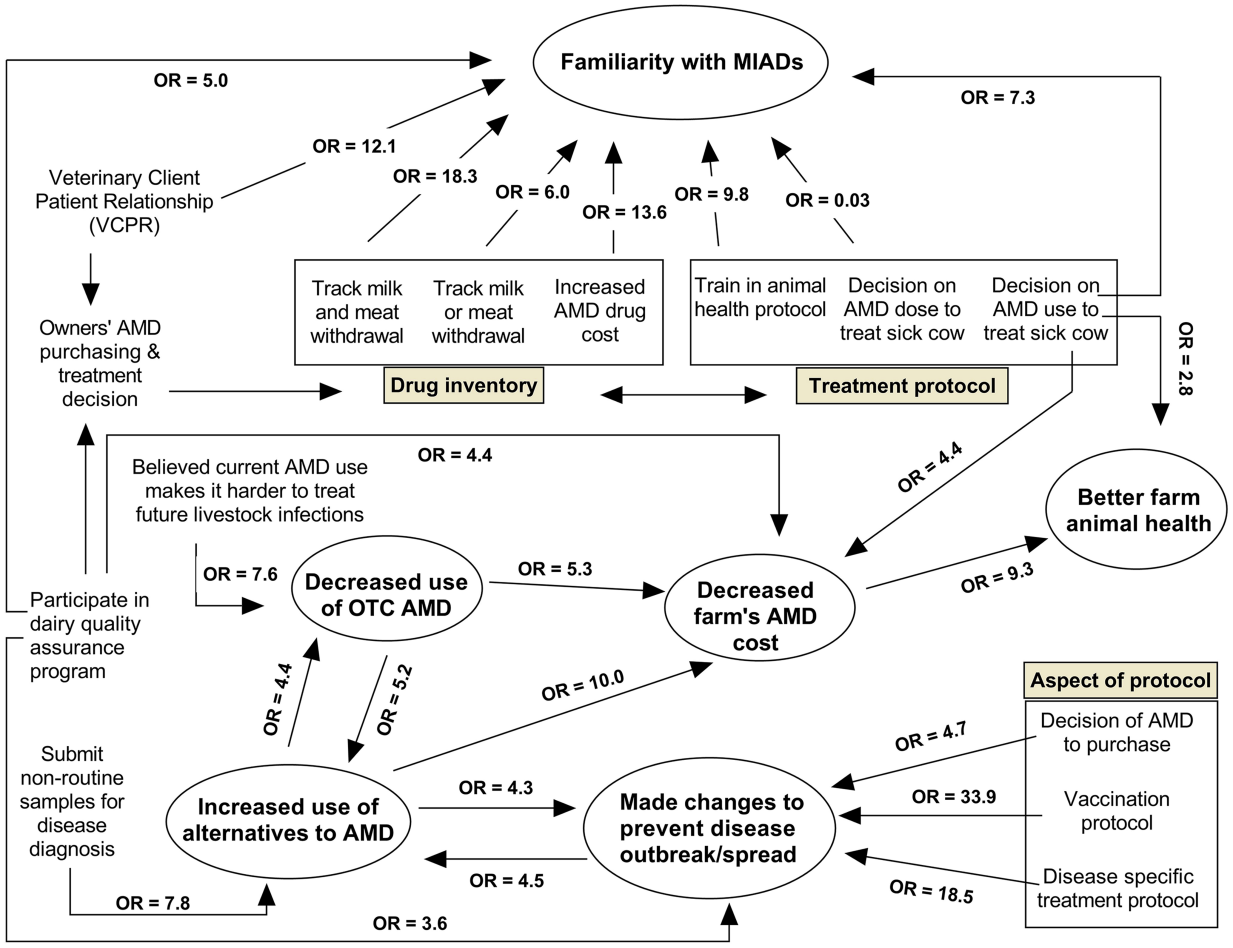
Web diagram showing the magnitude and direction of the associations between the six identified modeled outcomes and the predictor variables based on logistic regression models using survey responses from 132 conventional dairy producers in California. The six outcome variables are contained in the oval shaped bins. (MIAD = Medically important antimicrobial drugs; AMD = Antimicrobial drugs; OR = Odds ratio; OTC = over-the-counter).

Final models identifying predictors of the six outcomes, and the respective magnitude of each association, are depicted in Fig. 2. Tables 2–8 summarize the final logistic regression models for the six outcomes and their predictors. For all six models, the NCA and NSJV regions were combined due to limited sample size and contrasted to the GSCA region, with the latter as the reference. In all six models, region was not found to be significant. Similarly, breed as a predictor was not significant except in the model for farm AMD costs. Herd size, on the other hand, was a significant predictor in four of the six outcomes; familiarity with MIADs, use of alternatives to AMD, farm AMD costs, and the herd’s health status.

**Table 2:** Estimated coefficients and odds ratio from a multilevel mixed effects logistic regression model for the association between survey factors on AMD and familiarity with FDA^a^ “MIAD^b^” term in 99 respondents on conventional California dairies.

Variable	β coefficient	Robust SE	Odds ratio	95% CI		*P*-value
				Lower	Upper	
**Region**						
GSCA^c^	Referent					
NCA^d^ + NSJV^e^	– 0.34	0.73	0.70	0.16	2.96	0.636
**Herd size**						
<1,305	Referent					
1,305–3,500	– 1.77	0.77	0.16	0.03	0.76	0.021
>3,500	– 0.13	1.46	0.87	0.04	15.61	0.928
**Breed**						
Mixed/Other	Referent					
Holstein	– 1.07	0.77	0.34	0.07	1.53	0.161
Jersey	– 0.01	2.56	0.98	0.01	149.8	0.994
**Do you have a veterinarian-client-patient relationship (VCPR)**						
No	Referent					
Yes	2.49	1.19	12.12	1.17	125.07	0.036
**Who trained personnel on treatment protocol for sick cows**						
Dairy personnel only	Referent					
Veterinarian involved	2.28	0.90	9.83	1.66	58.22	0.012
**Who decides AMD^f^ used to treat sick cows**						
Dairy personnel only	Referent					
Veterinarian involved	1.98	0.93	7.28	1.16	45.46	0.033
**How are AMD doses for cows usually estimated**						
No Veterinarian involved	Referent					
Veterinarian involved	– 3.39	0.96	0.03	0.01	0.21	0.001
**Which AMD treatment information do you track/record**						
No Milk or Meat Withdrawal Interval	Referent					
Included Milk and Meat Withdrawal Interval	2.90	1.15	18.34	1.90	176.9	0.012
Included Milk or Meat Withdrawal Interval	1.79	1.05	6.02	0.76	47.60	0.089
**AMD cost since 1/1/2018 compared to 2017 and earlier**						
No change	Referent					
Increased	2.61	1.02	13.60	1.83	100.9	0.011
**Participate in local^g^ or national^h^ dairy quality assurance programs**						
No	Referent					
Yes	1.61	0.73	5.03	1.19	21.21	0.028

**Notes:**

a FDA = U.S. Food and Drug Administration.

b MIAD = Medically Important Antimicrobial Drug.

c GSCA = Greater southern California.

d NCA = Northern California.

e NSJV = Northern San Joaquin Valley.

f AMD = Antimicrobial drug.

g Local program = Creamery, On-farm training, Cooperate extension.

h National program = National Dairy FARM Program, Validus Dairy Animal Welfare Review.

Certification, California Dairy Quality Assurance Program.

**Table 3:** Estimated coefficients and odds ratios from a multilevel mixed effects logistic regression model for the association between survey factors and decreased use of AMD^a^ that were previously available over-the-counter (OTC) after January 2018 compared to 2017.

Variable	β coefficient	Robust SE	Odds ratio	95% CI		*P*-value
				Lower	Upper	
**Region**						
GSCA^b^	Referent					
NCA^c^ + NSJV^d^	–0.41	0.51	0.65	0.23	1.80	0.416
**Herd size**						
<1,305	Referent					
1,305-3,500	0.93	0.56	2.55	0.84	7.69	0.096
>3,500	–0.93	1.04	0.39	0.05	3.03	0.369
**Breed**						
Mixed/Other	Referent					
Holstein	–1.54	0.70	0.21	0.05	0.84	0.028
Jersey	–0.80	1.37	0.44	0.03	6.63	0.560
**Current AMD use makes it harder to treat future livestock infection**						
Strongly disagree	Referent					
Neutral	2.02	0.78	5.15	1.01	26.03	0.047
Strongly agree	1.63	0.82	7.56	1.63	35.07	0.010
**Use or increased use of alternatives to AMD since 2018**						
No	Referent					
Yes	1.48	0.69	4.42	1.12	17.44	0.034

**Notes:**

a AMD = Antimicrobial drug.

b GSCA = Greater southern California.

c NCA = Northern California.

d NSJV = Northern San Joaquin Valley.

The estimates were based on responses from 88 respondents from conventional California dairies that had used OTC AMDs prior to 2018.

**Table 4:** Estimated coefficients and odds ratios from a multilevel mixed effects logistic regression model for the association between survey factors and use or increased use of alternatives to AMD^a^ since January 2018.

Variable	β coefficient	Robust SE	Odds ratio	95% CI		*P*-value
				Lower	Upper	
**Region**						
GSCA^b^	Referent					
NCA^c^ + NSJV^d^	0.04	0.63	1.04	0.30	3.62	0.942
**Herd size**						
<1,305	Referent					
1,305–3,500	–2.24	0.85	0.10	0.01	0.56	0.009
>3,500	–0.73	1.11	0.47	0.05	4.23	0.507
**Breed**						
Mixed/Other	Referent					
Holstein	–0.96	0.72	0.38	0.09	1.60	0.188
Jersey	–0.16	1.49	0.84	0.04	15.78	0.910
**Submitted non-routine samples for infectious disease diagnosis in 2018**						
No	Referent					
Yes	2.04	0.75	7.75	1.76	34.09	0.007
**Decreased use of AMD^a^ previously available OTC^e^ in 2017**						
No	Referent					
Yes	1.65	0.65	5.21	1.45	18.72	0.011
**Made changes to prevent disease outbreak/spread since 2018**						
No	Referent					
Yes	1.50	0.65	4.49	1.25	16.10	0.021

**Notes:**

a AMD = Antimicrobial drug.

b GSCA = Greater southern California.

c NCA = Northern California.

d NSJV = Northern San Joaquin Valley.

e OTC = over-the-counter.

The estimates were based on responses from 110 respondents on conventional California dairies.

**Table 5:** Estimated coefficients and odds ratios from a multilevel mixed effects logistic regression model for the association between survey factors and whether the farm have made changes to prevent disease outbreak or spread since January 2018.

Variable	β coefficient	Robust SE	Odds ratio	95% CI		*P*-value
				Lower	Upper	
**Region**						
GSCA^a^	Referent					
NCA^b^ + NSJV^c^	–0.13	0.55	0.87	0.29	2.58	0.810
**Herd size**						
<1,305	Referent					
1,305-3,500	–1.02	0.63	0.35	0.10	1.25	0.109
>3,500	1.83	1.27	6.26	0.51	76.83	0.152
**Which aspects of animal health are protocols used**						
No protocols	Referent					
Included vaccination schedules	3.52	1.34	33.99	2.43	475.2	0.009
Disease-specific treatments	2.91	1.41	18.45	1.14	296.8	0.040
**Who decides injectable AMD^d^ to purchase**						
Dairy personnel only	Referent					
Veterinarian involved	1.55	0.60	4.71	1.43	15.51	0.011
**Use or increased use of alternatives to AMD since 2018**						
No	Referent					
Yes	1.45	0.68	4.29	1.11	16.48	0.034
**Participate in dairy quality assurance programs**						
No	Referent					
Yes	1.26	0.58	3.55	1.12	11.26	0.031

**Notes:**

a GSCA = Greater southern California.

b NCA = Northern California.

c NSJV = Northern San Joaquin Valley.

d AMD = Antimicrobial drug.

The estimates were based on responses from 99 respondents on conventional California dairies.

**Table 6:** Estimated coefficients and odds ratios from a multilevel mixed effects logistic regression model for the association between survey factors on AMD^a^ use and decreased farm’s AMD cost since January 2018 compared to 2017 and earlier.

Variable	β coefficient	Robust SE	Odds ratio	95% CI		*P*-value
				Lower	Upper	
**Region**						
GSCA^b^	Referent					
NCA^c^ + NSJV^d^	0.37	0.56	1.46	0.48	4.39	0.501
**Herd size**						
<1,305	Referent					
1,305–3,500	0.97	0.63	2.65	0.75	9.24	0.126
>3,500	2.93	1.22	18.78	1.69	208.2	0.017
**Breed**						
Mixed/Other	Referent					
Holstein	– 1.81	0.63	0.16	0.04	0.56	0.004
Jersey	– 2.44	1.42	0.08	0.01	1.42	0.087
**Use or increased use of alternatives to AMD^a^ since 2018**						
No	Referent					
Yes	2.30	0.76	10.02	2.22	45.14	0.003
**Changes made by farm regarding AMD previously available OTC^e^ since 2018 compared to 2017**						
No change	Referent					
Deceased use of AMD previously available OTC	1.66	0.60	5.31	1.61	17.44	0.006
**Who decides AMD use to treat sick cows**						
Dairy personnel only	Referent					
Veterinarian involved	1.49	0.59	4.44	1.38	14.32	0.012
**Participate in dairy quality assurance programs**						
No	Referent					
Yes	1.47	0.61	4.39	1.32	14.56	0.016

**Notes:**

a AMD = Antimicrobial drug.

b GSCA = Greater southern California.

c NCA = Northern California.

d NSJV = Northern San Joaquin Valley.

e OTC = over-the-counter.

The estimates were based on responses from 114 respondents on conventional California dairies.

**Table 7:** Estimated coefficients and odds ratios from a multilevel mixed effects logistic regression model for the association between survey factors and better farm animal health since January 2018 compared to 2017 and earlier.

Variable	β coefficient	Robust SE	Odds ratio	95% CI		*P*-value
				Lower	Upper	
**Region**						
GSCA^a^	Referent					
NCA^b^ + NSJV^c^	– 0.33	0.63	0.71	0.20	2.48	0.594
**Herd size**						
<1,305	Referent					
1,305–3,500	1.15	0.56	3.16	1.05	9.49	0.040
>3,500	0.80	1.12	2.23	0.24	20.44	0.476
**Breed**						
Mixed/Other	Referent					
Holstein	–0.17	0.63	0.84	0.24	2.90	0.786
Jersey	–0.66	1.43	0.51	0.03	8.56	0.643
**AMD^d^ cost since 1/1/2018 compared to 2017 and earlier**						
No change	Referent					
Decreased	2.22	0.68	9.28	2.40	35.91	0.001
**Who trained personnel on treatment protocol for sick cows**						
Dairy personnel only	Referent					
Veterinarian involved	1.03	0.53	2.81	0.99	7.97	0.051
**Changes made by farm regarding AMD previously available OTC^e^ since 2018 compared to 2017**						
No change	Referent					
Reduced use of AMD previously available OTC	–2.66	0.95	0.06	0.01	0.44	0.005
Region X Changes made regarding AMD previously available OTC since 2018 compared to 2017 (NCA + NSJV x Reduced use of AMD previously available OTC)	2.45	1.18	11.65	1.13	119.3	0.039

**Notes:**

a GSCA = Greater southern California.

b NCA = Northern California.

c NSJV = Northern San Joaquin Valley.

d AMD = Antimicrobial drug.

e OTC = over-the-counter.

The estimates were based on responses from 104 respondents on conventional California dairies.

**Table 8:** Estimated odds ratios for joint effects of region and changes made regarding use of AMD^a^ previously available over the counter (OTC^b^) on owner-reported better dairy cattle health status since January 2018 compared to 2017 or earlier.

Region × Changes regarding use of AMD^a^ previously available OTC^b^ (interaction)	Odds ratio	SE	95% CI		*P*-value
			Lower	Upper	
GSCA^c^ × No change	Referent				
GSCA x Reduced use of AMD previously available OTC	0.06	0.06	0.01	0.44	0.005
NCA^d^ + NSJV^e^ × No change	Referent				
NCA + NSJV × Reduced use of AMD previously available OTC	0.81	0.61	0.18	3.61	0.782
GSCA × Reduced use of AMD previously available OTC	Referent				
NCA + NSJV × Reduced use of AMD previously available OTC	8.29	7.96	1.26	54.4	0.027
GSCA × No change	Referent				
NCA + NSJV × No change	0.71	0.45	0.2	2.48	0.594

**Notes:**

a AMD = Antimicrobial drug.

b OTC = over-the-counter.

c GSCA = Greater southern California.

d NCA = Northern California.

e NSJV = Northern San Joaquin Valley.

The estimates were based on responses from 104 respondents on conventional California dairies.

#### Predictors concerning familiarity of dairies with the FDA “medically important antimicrobial drugs” (MIADs) term

Among the conventional dairies that completed the survey, a total of 86 respondents (69.9% ± 4.1) reported they were familiar with the FDA’s term ‘MIAD’, while 37 (30.1% ± 4.1) were not. Table 2 summarizes the final model for survey factors associated with familiarity of dairies with MIADs (R^2^ = 0.49; AIC = 90.25). Producers who reported having a VCPR with their herd veterinarian had greater odds (OR = 12.1) of being familiar with MIADs than those who reported they did not have VCPR. A VCPR is established when the client has authorized the licensed veterinarian to assume responsibility for making medical judgements and the need for medical treatment of the patient (including the prescription of AMDs when required) and the veterinarian has assumed that responsibility and has communicated with the client an appropriate course of therapy. For a valid VCPR, the veterinarian must be personally acquainted with the care of the animals by hands-on examination of the animal or by medically appropriate and timely visits to the premises where the animals are kept and have enough knowledge of the animals to give at least a general or preliminary diagnosis of the medical condition (*CCR § 2032.1*). Approximately 93.0% ± 2.2 of the dairies confirmed having a VCPR. Further analysis of the nine dairies who indicated they did not have a VCPR showed that eight included a veterinarian as a source they rely on for information about AMDs used to treat cows. In addition, one dairy indicated that they use other on-farm diagnostic techniques and procedures such as culture, auscultation, or lung ultrasound to guide treatment decisions with AMDs for cows on the dairy. These findings indicate compliance by almost all of survey respondents to the VCPR requirement, and even the producers who indicated not having a VCPR showed meaningful evidence of veterinary involvement in the health of their cattle. CA producers who did recognize the term VCPR and correlated it with their dairy herd health were at 12 times greater odds of being familiar with the term MIAD. Further outreach and continued education for dairy producers is required to update and complete the understanding of the requirement for a valid VCPR and what that entails.

Similarly, producers who included a veterinarian in the training of farm personnel on treatment protocols for sick cows had greater odds (OR = 9.8) of being familiar with MIADs compared to those who did not. Producers who included a veterinarian in the decision on what AMDs are used to treat sick cows had greater odds (OR = 7.3) of being familiar with MIADs compared to those who did not. Veterinarians’ knowledge about animal health, disease conditions, and indicators for AMD treatments and use may have directly informed producers of the term MIADs. Establishment of a valid VCPR and full engagement of the veterinarian in the discussions of dairy cattle health conditions by the producer may enhance the knowledge and familiarity of the producers about AMD stewardship and role of MIADs in maintaining the health of our cattle and prevention of AMR.

In contrast, producers who sought veterinarian input on how AMD doses are estimated had a 97% reduction in odds of being familiar with MIADs compared to those who did not. Only 33.1% ± 4.1 of the responding dairy producers confirmed they included a veterinarian in the decision of AMD dose estimation for cows, while the remaining 66.9% did not. Such producers may have delegated all AMD-related decisions to their veterinarians; and, thus, may have had fewer opportunities to learn about MIADs. Such producers may require more outreach on AMD use and stewardship given their greater reliance on their herd veterinarian for AMD use information, including dose estimation. Alternatively, producers may have initially consulted with their herd veterinarians to establish treatment protocols which included dosage guidance, and caregivers would regularly follow the protocol information for dosage amounts instead of directly involving the veterinarian for determining doses for specific disease conditions that commonly affect dairy cows. Furthermore, any extra-label drug use of medications in animals, including dosages different from those listed on the product labels, require a VCPR with the veterinarian’s authorization and directions for those extra-label uses.

Producers who participated in a dairy quality assurance program in the previous year had greater odds (OR = 5.0) of being familiar with MIADs compared with those who did not. Approximately 55.8% ± 4.5 of the responding dairy producers participated in dairy quality assurance programs, which included the National Dairy FARM Program (FARM), Validus Dairy Animal Welfare Review Certification (Validus), California Dairy Quality Assurance Program (CDQAP), Cooperative Extension trainings, creamery trainings, and on-farm trainings. The greater odds of familiarity with MIADs may be explained by the training and outreach such programs offer, which emphasize standards in animal health and welfare, written herd health plans, environmental and AMD stewardship, education regarding MIAD legislation, and judicious use of AMDs.

Producers who tracked both milk and meat withdrawal intervals had greater odds (OR = 18.3) of being familiar with MIADs compared with those who did not. Approximately 48.5% ± 4.3 of surveyed dairies specifically selected milk and meat withdrawal intervals as information they tracked, another 34.6% ± 4.1 confirmed tracking either milk or meat withdrawal intervals, and 16.9% ± 3.2 reported tracking neither milk nor meat withdrawal intervals. Producers who reported tracking withdrawal intervals for both milk and meat were largely located in the GSCA region (63.5% ± 6.0 vs. 31.8% ± 9.9; *P*-value = 0.010), involved a veterinarian in the training of dairy personnel on treatment protocols for sick cows (60.7% ± 6.5 vs. 0.0% ± 0.0; *P*-value = 0.001) and in determining treatment duration for AMD-treated cows (93.5% ± 3.1 vs. 63.6% ± 10.2; *P*-value = 0.001), as compared to those who did not track this information. In addition, more of these producers used a computer to track AMD treatments (84.1% ± 4.6 vs. 27.3% ± 9.4; *P*-value = 0.001) and AMD withdrawal periods (77.8% ± 5.2 vs. 27.3% ± 9.4; *P*-value = 0.001), and utilized bolus or injectable AMD (86.2% ± 4.5 vs. 61.1% ± 11.4; *P*-value = 0.019) for metritis treatment, compared to those who did not track.

Further analysis of a related question on the survey—“*Do you keep track of AMD withdrawal intervals (withholding periods) for treated cows?*”—showed that the remaining 16.9% ± 3.2 of producers who reported not tracking milk or meat withdrawal intervals confirmed they did, indeed, keep track of AMD withholding periods. The latter question included more information specifying withholding periods, a term that producers with such conflicting responses may have been more familiar with, as compared to the term ‘withdrawal interval’. These findings indicate compliance with tracking of AMD withdrawal intervals by all responding CA dairy producers.

Finally, producers who recorded increased AMD drug costs in 2018 compared to 2017 and earlier had greater odds (OR = 13.6) of being familiar with MIADs compared to those who did not. Approximately one-quarter (25.6% ± 3.8) of the responding dairy producers reported increased AMD cost since January 2018 compared to 2017 and earlier, while the remaining 74.4% ± 3.8 reported decrease or no change in AMD costs. A significantly higher percentage of producers who reported increased AMD cost since 2018 identified their herds as small sized (<1,305 milking cows) compared to those who reported decreased or no change (66.7% ± 8.2 vs. 45.8% ± 5.1; *P*-value = 0.038). Similarly, a significantly greater proportion of producers who reported increased AMD cost in 2018 were located in the NCA and GSCA compared to those who reported decreased or no change in the same regions (72.7% ± 7.7 vs. 52.1% ± 5.1; *P*-value = 0.038). Finally, 51.5% ± 8.6 of producers who reported increased AMD costs involved a veterinarian in estimating AMD doses for sick cows, as compared to 26.6% ± 4.5 for those who reported decreased or no change in AMD costs on their dairies (*P*-value = 0.008).

Producers’ familiarity with MIADs was mainly associated with producers having a valid VCPR for their dairies; involving a veterinarian in training personnel on sick cow treatment protocols and the decision on which AMD were used to treat sick cows; and producers’ participation in dairy quality assurance training programs in the last year. In addition, whether the producer tracked milk and meat withdrawal intervals, and whether the producer reported increased AMD costs since January 2018, were associated with producer familiarity with MIADs. To increase producer familiarity with MIADs, extension and outreach to dairy producers is necessary, providing them with training on what MIADs are and the stewardship practices that are required when using MIADs. Such education should be focused on producers who may not involve their veterinarian in the training of dairy personnel on sick cow treatment protocols and the decision on which AMD were used to treat sick cows, as well as those who have not participated in a dairy quality assurance training program in the last year.

#### Predictors concerning decreased use of AMD that were previously available over-the-counter (OTC) prior to January 2018

Among the conventional dairies who completed the survey, a total of 54 dairies (42.5% ± 4.3) reported that, in 2018, they decreased the use of AMDs that were previously available OTC, regardless of whether they used OTC AMD prior to 2018. Conversely, 73 dairies (57.5% ± 4.3) reported that, in 2018, they either increased or had no change in the use of AMDs previously available OTC, regardless of whether they used OTC AMD prior to 2018. For this analysis, we excluded survey respondents who did not use OTC AMD prior to 2018. After the exclusion of the dairies that did not use OTC AMD prior to 2018, 42 dairies (47.7% ± 5.3) reported decreased use of those AMDs that were previously available OTC, while 46 dairies (52.2% ± 4.3) reported increased or no change in those AMDs that were previously available OTC. Table 3 summarizes the final model for survey factors associated with decreased use of AMDs that were previously available OTC (R^2^ = 0.19; AIC = 115.92). Producers who indicated strong or neutral agreement to the AMR-related question “*Current antibiotic use practices in animal agriculture will make it harder to treat future livestock infections*” had higher odds (OR = 7.6; OR = 5.2; respectively) of decreased use of AMDs that were previously available OTC as compared to producers who strongly disagreed with the same AMR question. A higher percentage of the producers who agreed with the previous statement carried out vaccination of lactating cows against coliform mastitis compared to those who strongly disagreed with the same statement (94.6% ± 3.7 vs. 71.4% ± 12.1; *P*-value = 0.021), and producers with neutral agreement (93.3% ± 4.5 vs. 71.4% ± 12.1; *P*-value = 0.048). Furthermore, a significantly higher percentage of producers who indicated strong agreement to the AMD use practice question also: indicated harvesting colostrum from fresh cows to feed to newborn calves (100.0% ± 0.0 vs. 87.5% ± 8.2; *P*-value = 0.026), had used other on-farm diagnostic techniques to guide AMD treatment decisions for cows (86.1% ± 5.7 vs. 62.5% ± 12.1; *P*-value = 0.054), and ranked AMD use in preventing disease in high risk cows as very important (100.0% ± 0.0 vs. 81.3% ± 9.8; *P*-value = 0.006) compared to those who strongly disagreed. These findings indicate that producers who responded that current AMD use practices in animal agriculture will make it harder to treat future livestock infections also employed good livestock husbandry practices that include disease prevention and outbreak investigations to guide AMD treatment decisions, which may result in decreased AMD use on the dairy.

Producers who began using or increased their use of alternatives to AMD on their dairies in 2018 had higher odds (OR = 4.4) of decreased use of AMD that were previously available OTC as compared to producers who did not use alternatives to AMD. Further analysis of the producers who began using or increased use of alternatives to AMD in 2018 showed that a higher percentage of these producers used selective dry cow protocols for cows at the end of their lactation compared to those who neither began using nor increased use of alternatives to AMD in 2018 (42.1% ± 11.3 vs. 7.5% ± 3.2; *P*-value = 0.001). Similarly, a higher percentage of the producers who began using or increased use of alternatives to AMD in 2018 confirmed they had not used bolus or injectable AMDs to treat dairy cattle for pneumonia since January 2018 compared to those who did not use alternatives to AMD (11.8% ± 7.8 vs. 0.0% ± 0.0; *P*-value = 0.008). Such producers also submitted non-routine samples (e.g. milk culture, placenta, cow for necropsy) to a diagnostic lab for diagnosis of infectious diseases (83.3% ± 8.8 vs. 43.8% ± 6.2; *P*-value = 0.003), and confirmed they had made changes in management to prevent disease outbreak or spread compared to those who did not use AMD alternatives in 2018 (61.1% ± 11.5 vs. 26.9% ± 5.6; *P*-value = 0.007). Use of alternatives to antibiotics may have filled the gap in use of AMDs that were previously available OTC, as evident by the decrease use of AMDs for therapeutic purposes by producers who used or increased use of alternatives to AMD in 2018 as compared to those who do not.

In summary, producers who, in 2018, decreased their use of AMDs that were previously available OTC reported that they strongly agreed that current AMD use practices in animal agriculture will make it harder to treat future livestock infections and reported that they initiated or increased the use of alternatives to AMD on their dairies in 2018. Producers’ thoughts on the role of AMD on livestock infections and use of AMD alternatives were associated with disease prevention management practices on the dairies and use of disease diagnostics to inform selection and appropriate use of AMD.

#### Predictors concerning usage or increased use of alternatives to AMD since January 2018

Among the conventional dairies who completed the survey, a total of 25 respondents (20.3% ± 3.6) reported using or increased use of alternatives to AMD on their dairies since January 2018, while 98 respondents (79.7% ± 3.6) reported they did not use alternatives to AMD after January 2018. Table 4 summarizes the final model for survey factors associated with initiation or increased use of alternatives to AMD (R^2^ = 0.33; AIC = 90.89). Producers who reported submitting any non-routine samples such as milk culture, placental tissue, a cow for necropsy to a diagnostic laboratory for diagnosis of infectious diseases were at greater odds (OR = 7.8) to have reported that they initiated or increased use of alternatives to AMD in 2018 compared to those who did not submit non-routine samples to a diagnostic lab. Further analysis of producers who submitted non-routine samples for infectious disease diagnosis showed that a higher percentage of these producers had a large milking herd size (>1,305 milking cows) compared to those who did not submit non-routine samples to a laboratory for infectious disease diagnosis in 2018 (61.2% ± 5.9 vs. 36.2% ± 6.3; *P*-value = 0.005). Similarly, a higher percentage of the producers who submitted non-routine samples for infectious disease diagnosis in 2018 confirmed they carried out selective treatment of dry cows at dry-off compared to those who did not submit non-routine samples for diagnosis (22.4% ± 5.1 vs. 6.9% ± 3.3; *P*-value = 0.016). Such producers also confirmed keeping drug inventory logs for their dairies in greater proportion compared to those who did not submit non-routine samples for disease diagnosis (59.4% ± 6.1 vs. 40.4% ± 6.5; *P*-value = 0.037).

Producers who, in 2018, decreased the use of AMD that were previously available OTC had higher odds (OR = 5.2) of having initiated the use or increased use of alternatives to AMD on their dairies compared to producers who made no change or increased use of OTC AMD. A higher percentage of producers who decreased the use of AMD previously available OTC in 2018 also confirmed they carried out selective treatment of dry cows at dry-off compared to those who did not submit non-routine samples for diagnosis (22.2% ± 5.6 vs. 6.8% ± 2.9; *P*-value = 0.012).

In addition, producers who made changes in management to prevent disease outbreaks or spread on their dairies in 2018 had approximately 4.5 times greater odds of having started using or increased the use of alternatives to AMD on their dairies compared to those who did not. These three factors (1, submitted non-routine samples for infectious disease diagnosis; 2, decreased use of AMD; and 3, made changes to prevent disease outbreak/spread) found in the current study to be associated with use or increased use of alternatives to AMD were identified among the critical measures required to reduce the need for antimicrobial use in animal production (EMA & EFSA, 2017). Previous studies have shown that producers who are aware of infectious disease emergence, the impact of reliable diagnostics, and implementation of effective disease control measures were more prone to use or adopt disease preventive and control measures, such as vaccines, probiotics, phytochemicals, phages, immunomodulators, or teat sealants, on their dairies (Ekakoro et al., 2018; Kriebel et al., 2001).

#### Predictors concerning whether producers have made changes in management to prevent disease outbreak or spread since January 2018

Among the conventional dairies who completed the survey, a total of 44 respondents (36.1% ± 4.3) reported they had made changes in management to prevent disease outbreak or spread on their dairies since January 2018, while 78 respondents (63.9% ± 4.3) reported they had made no changes. Table 5 summarizes the final model for survey factors associated with the implementation of management changes to prevent disease outbreaks or spread (R^2^ = 0.31; AIC = 101.88). Producers who had written or computerized animal health protocols for vaccination schedules or disease-specific treatments had greater odds (OR = 33.9 and 18.5, respectively) of having made management changes to prevent disease outbreaks or spread since January 2018 compared to those who did not. The above findings emphasized the importance of having written or computerized animal health protocols on a dairy. The National Dairy FARM (Farmers Assuring Responsible Management) Animal Care Reference Manual describes a written protocol as a document containing instructions by a veterinarian on the management of various aspects of animal care on the dairy (https://nationaldairyfarm.com/wp-content/uploads/2018/10/Version-3-Manual-1.pdf). The protocols, which should be reviewed and updated annually or more often as needed, provide specific instructions to cow-side personnel for performing specific tasks. The protocols should include steps that define how the health of dairy animals will be monitored, how a health issue or disease will be identified, and what therapeutic procedure will be followed for animals identified as sick or having a health problem (Lewandowski, 2016). As a training tool, written protocols improve communication and work consistency, and inform adaptation in management as conditions change on the dairy (NMPF (National Milk Producers Federation), 2016). Dairy producers should be further educated on the benefits of having written or computerized animal health protocols for their dairies.

Similarly, producers who included a veterinarian in the decision on which AMD were purchased or stocked for treatment of adult cows were at greater odds (OR = 4.7) to have made management changes to prevent disease outbreaks or spread compared to those who did not. The herd veterinarian can be the professional with the knowledge of animal health and disease conditions on a specific dairy and, hence, is most capable of identifying the most successful and achievable herd-specific interventions to control and prevent diseases in the herd. Interaction between the veterinarian and dairy producers on the health care needs of their cattle is beneficial for the overall welfare and productivity of the herd (NMPF (National Milk Producers Federation), 2016). In addition, producers who used or increased use of alternatives to AMD on their dairies since January 2018 were at greater odds (OR = 4.2) of having made management changes to prevent disease spread or outbreaks compared to those who have not used any alternatives. The temporality of the latter association could not be confirmed due to the wording of the question and the cross-sectional nature of the study’s survey.

Finally, those producers who participated in dairy quality assurance programs in the last year had greater odds (OR = 3.6) of having made management changes to prevent disease outbreaks or spread compared to those who did not. Dairy quality assurance programs are voluntary programs that promote quality animal care practices, food safety and quality assurance, as well as enhance consumer confidence in the products from the dairies (CDRF (California Dairy Research Foundation), 2011). These programs, including CDQAP, FARM, cooperative extension, creamery-led programs, and on-farm training, provide training and standards for quality animal care to promote best management practices, environmental and AMD stewardship, and public health. The programs provide farmers with guidelines and tools to implement measures that contribute to good nutrition, health and husbandry, adequate treatments and treatment record keeping, housing and care of cows and youngstock, profitable marketing, and effective cattle handling (CDRF (California Dairy Research Foundation), 2011). The holistic nature of quality assurance programs may prepare participating producers to be proactive in identifying and making management changes for the wellbeing of their dairy cattle while promoting productivity. In summary, making management changes to prevent disease outbreak or spread post SB 27 was mainly associated with producers having written or computerized animal health protocols for their dairies, including involvement of a veterinarian in the decision on which AMDs were purchased or stocked for treatment of adult cows, using or increased use of AMD alternatives, and participation in dairy quality assurance programs.

#### Predictors concerning a farm’s AMD drug costs since January 2018 compared to 2017 and earlier

Changes in farm’s drug costs were dichotomized as “decreased AMD costs” versus “increased/no change” in AMD costs. Among the conventional dairies who completed the survey, a total of 39 respondents (30.2% ± 4.1) reported decreased farm’s AMD costs since January 2018, while 90 respondents (69.8% ± 4.1) reported increased or no change in farm’s AMD costs. Table 6 summarizes the final model for survey factors associated with decreased farm’s AMD costs (R^2^ = 0.36; AIC = 108.20). Producers who started using or increased their use of alternatives to AMD since January 2018 reported decreased farm AMD costs on their dairies since January 2018 compared to those who did not use alternatives to AMD (OR = 10.0). Similarly, producers who reported using less injectable and/or intramammary AMD that were previously available OTC after January 2018 (OR = 5.3) reported decreased farm AMD costs compared to those who did not. Amongst the alternatives to AMDs listed in the survey question were vitamins, minerals, herbal remedies, and vaccines. Several studies have highlighted the importance of vaccines and other alternative products in the prevention and control of infectious diseases in food-producing animals (Dubrovsky et al., 2019a, 2019b; Maier et al., 2019b; Paul-Pierre, 2009; Postma et al., 2015; Roth, 2011; Schukken et al., 2014). Increased use of alternatives to AMD in livestock production has been correlated with improved health status and a reduction in AMD use and AMR (Buchy et al., 2020; Hoelzer et al., 2018a, 2018b; Jansen, Knirsch & Anderson, 2018). It is not surprising to observe that producers who reported increased usage of alternatives to AMD post SB 27 implementation also reported decreased AMD drug costs on their dairies. Good management practices, use of vaccines (Buchy et al., 2020; Roth et al., 2003; Roth, 2011), use of teat sealants, and probiotic treatments help prevent diseases and the proliferation of pathogens, reduce AMD use due to fewer infections, prevent occurrence and spread of resistant strains, and improve animal welfare and public health (Ekakoro et al., 2018; Hoelzer et al., 2018a). Increased use of alternatives to AMD may improve the health condition of dairy cattle and reduce the number of animals who require AMD treatment, thereby resulting in an overall reduction in AMD costs on the dairy. Similarly, increased use of alternatives to AMD on the dairies may have replaced previously available OTC AMD and may be less expensive compared to OTC AMD.

Likewise, producers who included a veterinarian in the decision on which AMD were used to treat a sick cow reported decreased AMD costs compared to those who did not (OR = 4.4). In addition, producers who participated in dairy quality assurance programs in the previous year reported decreased AMD costs compared to those who did not (OR = 4.3). Inclusion of a veterinarian in animal health decisions, specifically, on the choice of AMD used in the treatment of sick cows, as well as participation in a dairy quality assurance program may have all promoted excellent dairy cattle health which may be evident from the decrease in AMD costs. Overall, decreased farm’s AMD costs post SB 27 implementation were mainly associated with initiation or increased use of alternatives to AMD, decreased use of AMD that were previously available OTC, involving a veterinarian in the decision on which AMD were used to treat sick cows, and producers’ participation in dairy quality assurance programs.

#### Predictors concerning a farm’s animal health status since January 2018 compared to 2017 and earlier

Respondents’ reports about farm animal health status since SB 27 implementation as an outcome was dichotomized as “better animal health” vs. “worse/no change”. Among the conventional dairies who completed the survey, a total of 39 respondents (32.2% ± 4.2) reported better animal health since January 2018 as compared to 2017 and earlier, while 82 respondents (67.8% ± 4.1) reported worse or no change in the farm’s animal health. Table 7 summarizes the final model for survey factors associated with better animal health on the farms (R^2^ = 0.24; AIC = 118.83). Producers who reported a decrease in their farm’s AMD costs since January 2018 compared to 2017 and earlier reported better animal health status on their dairies as compared to those who reported no change in farm’s AMD costs (OR = 9.2). Producers who included a veterinarian in the decision of which AMD were used to treat sick cows reported better animal health status on their dairies compared to those who did not (OR = 2.8). Such factors constitute good management and husbandry practices, hence, their link to better animal health status on dairies (CDFA (California Department of Food and Agriculture), 2019b, 2018b).

Dairies with 1,305 to 3,500 average number of milking cows reported better animal health (OR = 3.1, 95% CI [1.0–9.4], *P* = 0.04) compared to those with <1,305 average milking cows. Decreased use of AMD that were previously available OTC was also associated with better animal health status post SB 27 implementation but varied by (interaction) region (Table 8). Respondents located in NCA and NSJV, who reported decreased use, in 2018, of AMD previously available OTC, reported better animal health status in their herds (OR = 8.3, 95% CI [1.3–54.4], *P* = 0.02) compared to similar GSCA producers who also reported decreased use of AMD previously available OTC. Producers in GSCA who reported decreased use of AMD previously available OTC since 2018 also reported worse animal health on their dairies (OR = 0.06; 95% CI: [0.01–0.44]) compared to producers in same region who reported no change in use of AMD previously available OTC, representing 16.7% (5/30) of respondents. The five respondents in GSCA who reported no change in use of AMD previously available OTC had RHA lower than 10,660 kg/cow, and also had not used alternatives to AMD since January 2018.

### Machine learning classification models

#### Producers’ perceptions regarding the importance of antimicrobial drug (AMD) use stewardship practices

**Figure 3:**
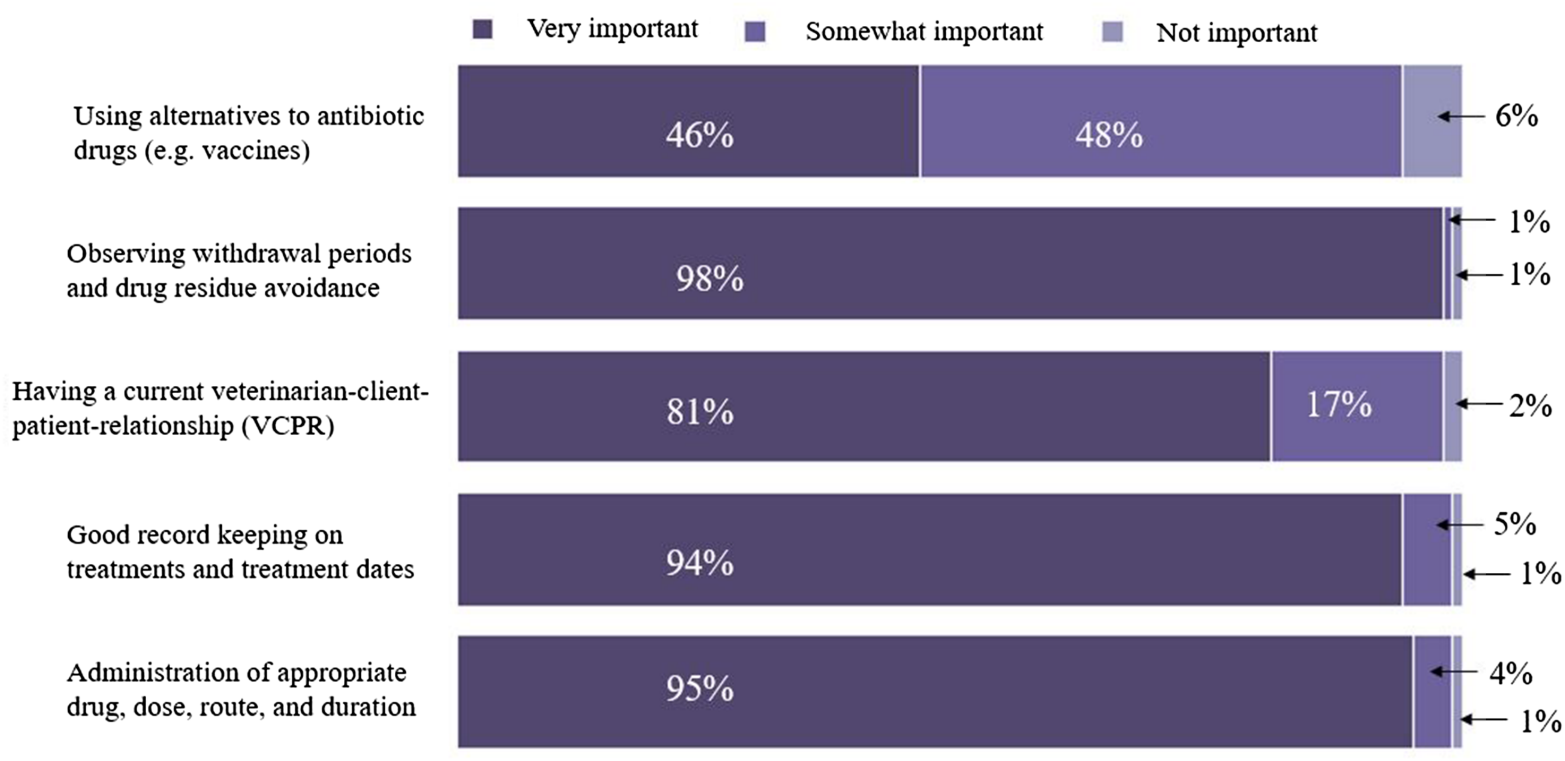
Distribution of responses to questions regarding how important dairy producers ranked five antimicrobial drug use stewardship indices. The plot summarized responses from 132 conventional dairy producers in California.

The distribution of conventional dairy producers’ responses to the ranking of five AMD use stewardship questions are presented in Fig. 3. Additionally, 36% (47/132), 44% (58/132), 16% (21/132), 5% (6/132), and 0% respondents ranked all 5, at least 4, 3, 2, or 1, respectively, of the AMD use stewardship practices as “very” or “somewhat important”. Approximately four-fifths (79.5%, 105 out of 132) of respondent’s dairies were classified as having “good-excellent” AMD use stewardship practices based on a score of ≥4. The remaining one-fifth (20.5%) were classified as having “limited-moderate” AMD use stewardship practices based on a score of ≤3. A descriptive analysis based on the classification of AMD use stewardship practices showed a significantly higher percentage of respondents classified as having limited-moderate stewardship practices included producers with smaller herd size (<1,305 milking cows) dairies (27.9% ± 5.4 vs. 12.5% ± 4.1; *P*-value = 0.027), those whose annual RHA was <10,600 kg/cow (29.3% ± 7.1 vs. 14.1% ± 3.9; *P*-value = 0.046 ), and those who did not use written/computerized animal health protocols for their dairies (71.0% ± 8.1 vs. 5.0% ± 2.1; *P*-value =0.001) compared to those with good-excellent practices (Table 9). In addition, a higher percentage of producers classified as having limited-moderate stewardship practices did not keep drug inventory logs (36.8% ± 5.8 vs. 3.1% ± 2.1; *P*-value = 0.001), did not track AMD withdrawal interval (100.0% ± 0.0 vs. 18.0% ± 3.3; *P*-value = 0.001), did not vaccinate lactating cows against mastitis (52.4% ± 10.8 vs. 14.4% ± 3.3; *P*-value = 0.001), did not participate in animal welfare audit programs in the past year (52.6% ± 11.4 vs. 15.0% ± 3.3; *P*-value = 0.001), and were not aware that MIADs required veterinary prescription since January 2018 (60.0% ± 21.9 vs. 18.9% ± 3.4; *P*-value = 0.001) compared to those with good-excellent practices.

**Table 9:** Descriptive analysis of producers classified as having limited-moderate antimicrobial drug use stewardship knowledge based on survey responses in 132 conventional California dairies.

Characteristics	Number of respondents	Respondents with limited-moderate knowledge	Proportion with limited-moderate knowledge (%)	Standard error (%)	95% confidence interval (%)		*P*-value
					Lower	Upper	
**Number of milking cows (herd size)**							
<1,305	68	19	27.9	5.4	17.3	38.6	0.027
>=1,305	64	8	12.5	4.1	4.4	20.6	
**Rolling herd average (Kg/cow)**							
<10,660	41	12	29.3	7.1	15.3	43.2	0.046
>=10,660	78	11	14.1	3.9	6.4	21.8	
**Use written/computerized health protocol**							
No	31	22	71.0	8.1	54.9	86.9	0.001
Yes	101	5	5.0	2.1	0.7	9.1	
**Have drug inventory log**							
No	68	25	36.8	5.8	25.3	48.2	0.001
Yes	64	2	3.1	2.1	0.0	7.3	
**Track antibiotic withdrawal**							
No	4	4	100.0	0.0	100.0	100.0	0.001
Yes	128	23	18.0	3.3	11.3	24.6	
**Vaccinate lactating cows against mastitis**							
No	22	11	52.4	10.8	31.0	73.7	0.001
Yes	111	16	14.4	3.3	7.8	20.9	
**Participate in animal welfare audit programs**							
No	19	10	52.6	11.4	30.1	75.0	0.001
Yes	113	17	15.0	3.3	8.4	21.6	
**Aware MIADs require prescription**							
No	5	3	60.0	21.9	17.0	100.0	0.025
Yes	127	24	18.9	3.4	12.0	25.7	

**Note:**

MIADs = Medically important antimicrobial drugs.

**Figure 4:**
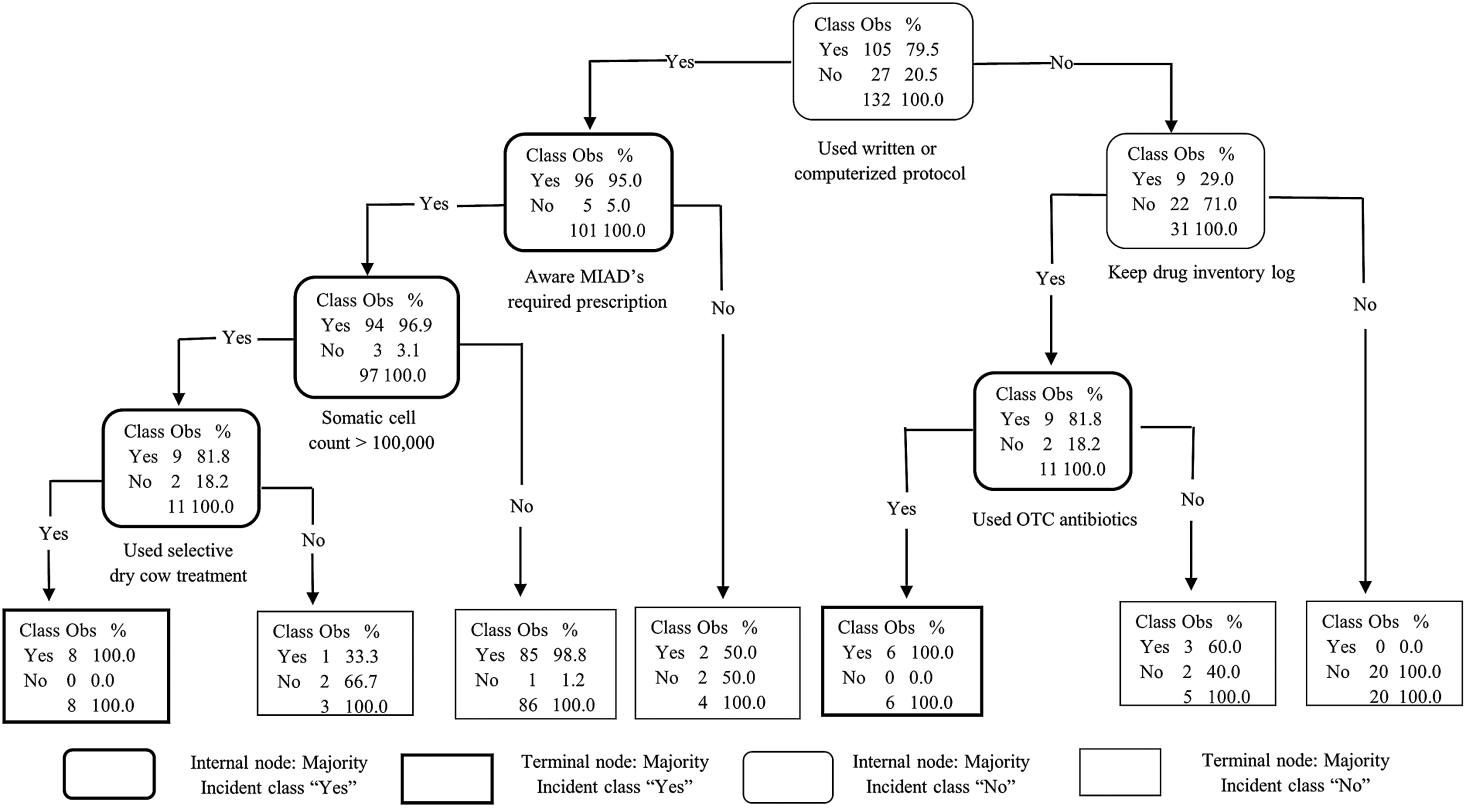
Decision tree analysis of 132 California conventional dairy producers’ responses on 5 antimicrobial stewardship indices. The indices assessed were: (1) administration of appropriate antimicrobial drug (AMD), dose, route, and duration; (2) good record keeping on treatments and treatment dates; (3) having a current veterinarian-client-patient relationship (VCPR); (4) observing withdrawal periods and drug residue avoidance; and (5) using alternatives to AMD (e.g. vaccines, supplements). Producers who identified at least 4 of the 5 indices as somewhat or very important were classified as having “good-excellent” knowledge of antimicrobial stewardship practices and the rest as having “limited-moderate” knowledge. (Obs = observations; MIADs = Medically important antimicrobial drugs; OTC = over-the-counter).

**Figure 5:**
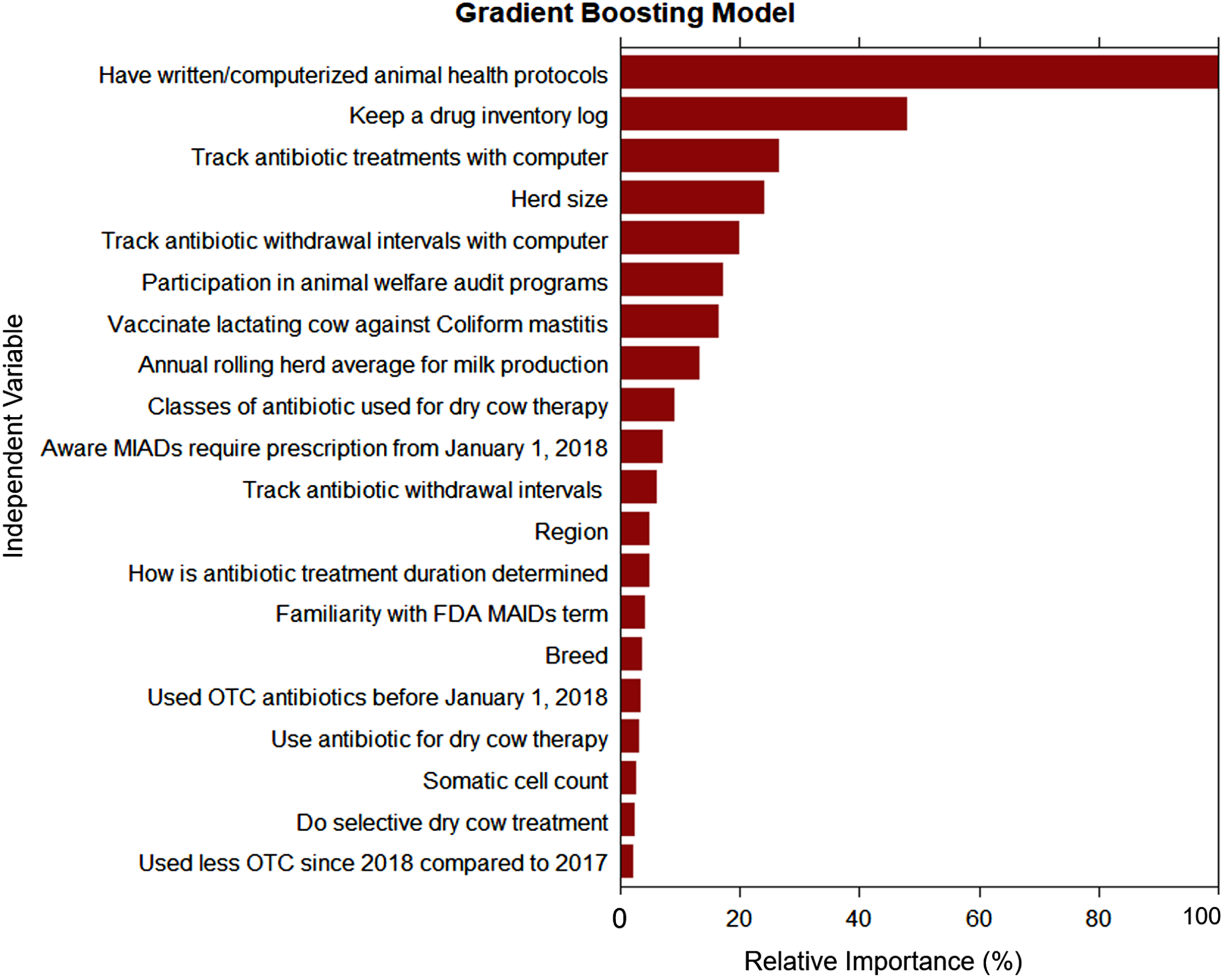
Ranking of variable relative importance for predicting antimicrobial drug use stewardship knowledge based on responses from 132 conventional dairy producers in California.

The goal of our classification models was to identify the key traits or factors that can identify producers with good-excellent AMD use and stewardship practices (outcome). For each classification algorithm, the final model was selected targeting the highest specificity (lowest false positive rate) in an effort to prioritize identifying dairies with limited-moderate AMD stewardship practices that may benefit from training on AMD use stewardship practices. This strategy necessarily resulted in a reduction of sensitivity (moderate false negative rate) which may have misclassified some dairies with “good-excellent” AMD stewardship practices as “limited-moderate” practices, resulting in potentially un-needed training for those dairies. Additional un-needed training to some producers with good-excellent AMD stewardship practices is acceptable, provided outreach is available for most or all dairies with a need for outreach for AMD use stewardship practice trainings. All three models agreed in their ranking of the two most important predictors for “good-excellent” AMD use stewardship practices for California’s commercial dairy producers: use of written or computerized animal health protocols and keeping a drug inventory log. The DT model derived from recursive partitioning of the producers AMD use stewardship practices is summarized in Fig. 4. The DT model showed that the variables: use written or computerized animal health protocols, keep a drug inventory log, and aware that MIADs required prescription were important predictors for classifying dairy producers’ AMD stewardship practices. However, the GB model had the highest specificity (100%) and the best prediction of all three algorithms for classifying the producers’ AMD stewardship practices (Table 10). The GB model ranking identified the top six most important variables as: use written or computerized animal health protocols; keep a drug inventory log; aware that MIADs required a prescription; veterinarian input in determination of treatment durations; use selective dry cow treatment; and participation in animal welfare audit programs (Fig. 5). A comparison of model rankings for all three algorithms is presented in Appendix 1.

**Table 10:** Results for the average performance of 3 classification models for the association between good-excellent antimicrobial drug use stewardship practices and survey responses in 132 conventional California dairies.

Model performance	Model		
	Decision tree	Random forest	Gradient boosting
Specificity	85.2	92.6	100.0
Sensitivity	66.7	69.5	99.0
Precision^1^	94.6	97.3	100.0
F1 score^2^	78.2	81.1	99.5
Classification accuracy^3^	70.5	74.2	99.2
Balanced accuracy^4^	75.9	81.1	99.5
Matthew’s correlation coefficient^5^	0.42	0.51	0.98
Area under ROC^6^ Curve (AUC)	0.85	0.92	0.99

**Notes:**

1 Positive predictive value (True positive / (True positive + False positive)).

2 Harmonic average of sensitivity and positive predictive value (2 × sensitivity × positive predictive value )/(sensitivity + positive predictive value).

3 Percent of correct predictions.

4 Average of sensitivity and specificity.

5 Correlation coefficient between observed and predicted binary classifications between −1 and + 1 with +1 representing a perfect prediction.

6 Receiver operating characteristic.

Using written or computerized animal health protocols is a good standard practice critical to the promotion and maintenance of animal health and welfare and the protection of public health (Apley, 2015; McGuirk, 2008; Pappaioanou, 2004). For the producers who were classified as having “limited-moderate” AMD use stewardship practices, 81.5% (22/27) did not use written or computerized animal health protocols on their dairies, and 70.4% (19/27) had herd sizes <1,305 milking cows. Although a majority of the respondent dairies classified as having “limited-moderate” stewardship practices reported having a VCPR (100%), consulted a veterinarian as source for AMD information (96.3%) or for determination of AMD treatment duration (76.9%), and were aware of the prescription requirement for MAIDs (88.9%), a majority of these dairies did not keep a drug inventory log (92.6%), used no computer in tracking of AMD treatments (66.7%) or withdrawal periods for treated cows (69.2%). The absence of computerized protocols on dairies with “limited-moderate” stewardship practices may be related to the small herd size (median = 668, 5^th^ percentile = 200 and 95^th^ percentile = 1,630 milking cows) on a majority of these dairies. Overall, a higher percentage of respondents with small herd sizes (<1,305 milking cows) documented cows’ events on paper notes, white boards, or commit such events to memory compared with those with >1,305 milking cows (74.4% ± 6.6 vs. 40.4% ± 5.2; *P*-value = 0.001). Education and outreach should be extended to small-and medium-sized dairies on the importance of having properly documented, written or computerized health protocols, as well as drug inventory logs for their dairies to facilitate adequate record keeping, which is essential for excellence in antimicrobial stewardship (CDFA (California Department of Food and Agriculture), 2019b, 2018b).

### Limitations of the study

Limitations of this study include its reliance on responses obtained through a survey mailed to CA licensed Grade A dairy producers, which may be subject to recall and/or reporting bias. Similarly, respondents may have interpreted the questions differently, as in the case of some producer’s understanding of the term AMD withholding periods versus withdrawal intervals. As this survey was self-reported, the AMD usage or treatment practices and the health status of the cows on respondent dairies could not be ascertained. Furthermore, actual AMD drug use, and resistance pattern were not measured in this study. Antimicrobial drug use stewardship practices were ranked by producers and not on the direct observation and measure of the actual practices on the dairies. Finally, the antimicrobial stewardship practices cannot be characterized by the current survey’s statistical analysis alone. Hence, further studies are needed to directly measure the associations between AMD use treatment practices and stewardship practices based on evaluation of management and production records.

## Conclusions

The current study modeled the association between management practices on conventional dairies and producers’ familiarity with MIAD, change in use of AMDs previously available OTC, use of alternatives to AMD, changes to prevent disease outbreaks, changes in AMD costs and animal health status on conventional dairies post SB 27. In addition, we adopted a machine learning approach to determine important predictors of good-excellent antimicrobial stewardship practices among CA conventional dairy producers. The main findings of our research can be summarized as follows:

1. For the model exploring the association between management practices and producers’ familiarity with MIAD, having a VCPR, involving veterinarian in training on treatment protocols and decisions, tracking AMD treatment information, and participation in dairy quality assurance programs were the significant predictors.
2. In the model exploring decreased use of AMDs previously available OTC, responses that current AMD use practices in animal agriculture will make it harder to treat future livestock infections, and initiation or increased use of alternatives to AMD on dairies post SB 27 were significant predictors.
3. Management factors associated with initiation or increased use of alternatives to AMD post SB 27 were decreased use of AMDs that were previously available OTC, dairies submitting non-routine samples to a laboratory for disease diagnosis, as well as making management changes to prevent disease outbreak or spread.
4. For the model exploring the association between management practices and whether producers’ made changes to prevent disease outbreaks post SB 27, having written/computerized animal health protocols, including veterinarian in AMD treatment decisions, increasing use of alternatives to AMD, and participating in dairy quality assurance program were the significant predictors.
5. For the model exploring the association between management practices and reported decrease in farm’s AMD cost post SB 27, initiation or increased use of alternatives to AMD post SB 27, decreased use of AMD that were previously available OTC, including a veterinarian in AMD treatment decisions, and participating in a dairy quality assurance program were the significant predictors.
6. Management factors associated with better animal health post SB 27 were reported decrease in farm’s AMD cost post SB 27, including a veterinarian in AMD treatment decisions, and decreased use of AMDs that were previously available OTC.
7. Finally, using written or computerized animal health protocols, keeping a drug inventory log, awareness that since SB 27 the use of MIADs required a prescription, and involving a veterinarian in the determination of AMD treatment duration were the most important characteristics of “good-excellent” AMD stewardship practices among conventional CA dairy producers identified in this study.

Our survey findings benchmark the CA dairy industry’s antimicrobial stewardship practices during the first year after implementation of SB 27. Producers will benefit from extension outreach efforts that incorporate the findings of this survey by further highlighting the significance of these management practices and encouraging those that are associated with judicious AMD use and stewardship practices on CA dairies.

## Supplemental Information

10.7717/peerj.11596/supp-1 Supplemental Information 1 Comparison of rankings of variable relative importance for predicting antimicrobial drug use stewardship knowledge based on responses from 132 conventional dairy producers in California. The rankings were based on three classification algorithms (Decision tree, Random forest, and Gradient boosting)

10.7717/peerj.11596/supp-2 Supplemental Information 2 2018 Survey of Antibiotic Drug Use in California Dairy Cows.

## Additional Information and Declarations

### Data Availability

The following information was supplied regarding data availability: This study was sponsored by the California Department of Food and Agriculture and is subject to California Food and Agriculture Code (FAC) Sections 14400 to 14408. FAC section 14407 requires that data collected be held confidential to prevent individual identification of a farm or business; as such, raw data from this study is not able to be shared.

## References

[ref-1] Aly SS, Anderson RJ, Adaska JM, Jiang J, Gardner IA (2010). Association between Mycobacterium avium subspecies paratuberculosis infection and milk production in two California dairies. Journal of Dairy Science.

[ref-2] Amrine DE, McLellan JG, White BJ, Larson RL, Renter DG, Sanderson M (2019). Evaluation of three classification models to predict risk class of cattle cohorts developing bovine respiratory disease within the first 14 days on feed using on-arrival and/or pre-arrival information. Computers and Electronics in Agriculture.

[ref-3] Apley MD (2015). Feedlot pharmaceutical documentation: protocols, prescriptions, and veterinary feed directives. Veterinary Clinics of North America: Food Animal Practice.

[ref-4] Breiman L (2001). Random forests. Machine Learning.

[ref-5] Buchy P, Ascioglu S, Buisson Y, Datta S, Nissen M, Tambyah PA, Vong S (2020). Impact of vaccines on antimicrobial resistance. International Journal of Infectious Diseases.

[ref-6] CA (California) Senate (2015). SB-27 livestock: use of antimicrobial drugs.

[ref-7] CDC (U.S. Centers for Disease Control and Prevention) (2019). Antibiotic resistance threats in the United States, 2019.

[ref-8] CDFA (California Department of Food and Agriculture) (2019a). Antimicrobial use and stewardship program annual report 2019.

[ref-9] CDFA (California Department of Food and Agriculture) (2019b). Guidelines for judicious use of antimicrobials in livestock.

[ref-10] CDFA (California Department of Food and Agriculture) (2018a). California agricultural statistics review 2017–2018.

[ref-11] CDFA (California Department of Food and Agriculture) (2018b). Principles of antimicrobial stewardship.

[ref-12] CDRF (California Dairy Research Foundation) (2011). California dairy quality assurance program. http://cdrf.org/home/checkoff-investments/cdqap/.

[ref-13] Chen T, Guestrin C (2016). XGBoost: a scalable tree boosting system.

[ref-14] Daniel W (2005). Estimation. Biostatistics: A Foundation for Analysis in the Health Sciences.

[ref-47] Davies R, Wales A (2019). Antimicrobial resistance on farms: A review including biosecurity and the potential role of disinfectants in resistance selection. Comprehensive Reviews in Food Science and Food Safety.

[ref-15] Dubrovsky SA, Van Eenennaam AL, Karle BM, Rossitto PV, Lehenbauer TW, Aly SS (2019a). Epidemiology of bovine respiratory disease (BRD) in preweaned calves on California dairies: the BRD 10 K study. Journal of Dairy Science.

[ref-16] Dubrovsky SA, Van Eenennaam AL, Karle BM, Rossitto PV, Lehenbauer TW, Aly SS (2019b). Bovine respiratory disease (BRD) cause-specific and overall mortality in preweaned calves on California dairies: the BRD 10 K study. Journal of Dairy Science.

[ref-17] Ekakoro JE, Caldwell M, Strand EB, Okafor CC (2018). Drivers of antimicrobial use practices among tennessee dairy cattle producers. Veterinary Medicine International.

[ref-50] Ekong PS, Abdelfattah EM, Okello E, Williams DR, Lehenbauer TW, Karle BM, Rowe JD, Marshall ES, Aly SS (2021). 2018 Survey of antimicrobial drug use and stewardship practices in adult cows on California dairies: post-Senate Bill 27. PeerJ.

[ref-18] EMA and EFSA (2017). EMA and EFSA joint scientific opinion on measures to reduce the need to use antimicrobial agents in animal husbandry in the European Union, and the resulting impacts on food safety (RONAFA). EFSA Journal.

[ref-19] FAC (Food and Agricultural Code) (2015). Livestock: use of antimicrobial drugs [14400–14408].

[ref-49] FDA (U.S Food and Drug Administration) (2020). 2019 Summary report on antimicrobials sold or distributed for use in food-producing animals. https://www.fda.gov/media/144427/download.

[ref-20] Fei Y, Gao K, Hu J, Tu J, Li W, Wang W, Zong G (2017). Predicting the incidence of portosplenomesenteric vein thrombosis in patients with acute pancreatitis using classification and regression tree algorithm. Journal of Critical Care.

[ref-21] Friedman JH (2002). Stochastic gradient boosting. Computational Statistics & Data Analysis.

[ref-22] Hoelzer K, Bielke L, Blake DP, Cox E, Cutting SM, Devriendt B, Erlacher-Vindel E, Goossens E, Karaca K, Lemiere S, Metzner M, Raicek M, Collell Suriñach M, Wong NM, Gay C, Van Immerseel F (2018a). Vaccines as alternatives to antibiotics for food producing animals. part 1: challenges and needs. Veterinary Research.

[ref-23] Hoelzer K, Bielke L, Blake DP, Cox E, Cutting SM, Devriendt B, Erlacher-Vindel E, Goossens E, Karaca K, Lemiere S, Metzner M, Raicek M, Collell Suriñach M, Wong NM, Gay C, Van Immerseel F (2018b). Vaccines as alternatives to antibiotics for food producing animals—part 2: new approaches and potential solutions. Veterinary Research.

[ref-24] Jansen KU, Knirsch C, Anderson AS (2018). The role of vaccines in preventing bacterial antimicrobial resistance. Nature Medicine.

[ref-25] Jayarao B, Almeida R, Oliver SP (2019). Antimicrobial resistance on dairy farms. Foodborne Pathogens and Disease.

[ref-26] Kriebel D, Tickner J, Epstein P, Lemons J, Levins R, Loechler EL, Quinn M, Rudel R, Schettler T, Stoto M (2001). The precautionary principle in environmental science. Environmental Health Perspectives.

[ref-27] Lewandowski R (2016). Dairy herd health protocols.

[ref-28] Love WJ, Lehenbauer TW, Karle BM, Hulbert LE, Anderson RJ, Van Eenennaam AL, Farver TB, Aly SS (2016). Survey of management practices related to bovine respiratory disease in preweaned calves on California dairies. Journal of Dairy Science.

[ref-29] Maier GU, Love WJ, Karle BM, Dubrovsky SA, Williams DR, Champagne JD, Anderson RJ, Rowe JD, Lehenbauer TW, Van Eenennaam AL, Aly SS (2019a). Management factors associated with bovine respiratory disease in preweaned calves on California dairies: the BRD 100 study. Journal of Dairy Science.

[ref-30] Maier GU, Rowe JD, Lehenbauer TW, Karle BM, Williams DR, Champagne JD, Aly SS (2019b). Development of a clinical scoring system for bovine respiratory disease in weaned dairy calves. Journal of Dairy Science.

[ref-31] McGuirk SM (2008). Disease management of dairy calves and heifers. Veterinary Clinics of North America: Food Animal Practice.

[ref-32] NMPF (National Milk Producers Federation) (2016). The 2017 national dairy FARM (farmers assuring responsible management) animal care reference manual. https://nationaldairyfarm.com/wp-content/uploads/2018/10/Version-3-Manual-1.pdf.

[ref-33] Oliver SP, Murinda SE, Jayarao BM (2011). Impact of antibiotic use in adult dairy cows on antimicrobial resistance of veterinary and human pathogens: a comprehensive review. Foodborne Pathogens and Disease.

[ref-34] Pappaioanou M (2004). Veterinary medicine protecting and promoting the public’s health and well-being. Preventive Veterinary Medicine.

[ref-35] Paul-Pierre P (2009). Emerging diseases, zoonoses and vaccines to control them. Vaccine.

[ref-36] Postma M, Stärk KDC, Sjölund M, Backhans A, Beilage EG, Lösken S, Belloc C, Collineau L, Iten D, Visschers V, Nielsen EO, Dewulf J (2015). Alternatives to the use of antimicrobial agents in pig production: a multi-country expert-ranking of perceived effectiveness, feasibility and return on investment. Preventive Veterinary Medicine.

[ref-37] Rikin S, Glatt K, Simpson P, Cao Y, Anene-Maidoh O, Willis E (2015). Factors associated with increased reading frequency in children exposed to reach out and read. Academic Pediatrics.

[ref-38] Roth F, Zinsstag J, Orkhon D, Chimed-Ochir G, Hutton G, Cosivi O, Carrin G, Otte J (2003). Human health benefits from livestock vaccination for brucellosis: case study. Bulletin of the World Health Organization.

[ref-39] Roth JA (2011). Veterinary vaccines and their importance to animal health and public health. Procedia in Vaccinology.

[ref-40] Schukken YH, Bronzo V, Locatelli C, Pollera C, Rota N, Casula A, Testa F, Scaccabarozzi L, March R, Zalduendo D, Guix R, Moroni P (2014). Efficacy of vaccination on Staphylococcus aureus and coagulase-negative staphylococci intramammary infection dynamics in 2 dairy herds. Journal of Dairy Science.

[ref-48] Stelling J, Read JS, Fritch W, O’Brien TF, Peters R, Clark A, Bokhari M, Lion M, Katwa P, Kelso P (2020). Surveillance of antimicrobial resistance and evolving microbial populations in Vermont: 2011–2018. Expert Review of Anti-infective Therapy.

[ref-41] Strobl C, Malley J, Tutz G (2009). An introduction to recursive partitioning: rationale, application, and characteristics of classification and regression trees, bagging, and random forests. Psychological Methods.

[ref-42] Tang KL, Caffrey NP, Nóbrega DB, Cork SC, Ronksley PE, Barkema HW, Polachek AJ, Ganshorn H, Sharma N, Kellner JD, Ghali WA (2017). Restricting the use of antibiotics in food-producing animals and its associations with antibiotic resistance in food-producing animals and human beings: a systematic review and meta-analysis. The Lancet Planetary Health.

[ref-44] WHO (World Health Organization) (2015). Global action plan on antimicrobial resistance [WWW document]. http://www.who.int/antimicrobial-resistance/publications/global-action-plan/en/.

[ref-45] Zhang S (2012). Decision tree classifiers sensitive to heterogeneous costs. Journal of Systems and Software.

[ref-46] Zhang Z, Zhao Y, Canes A, Steinberg D, Lyashevska O (2019). Written on behalf of AME big-data clinical trial collaborative group, predictive analytics with gradient boosting in clinical medicine. Annals of Translational Medicine.

