# Supplementary figures and images for: 2018 Survey of factors associated with antimicrobial drug use and stewardship practices in adult cows on conventional California dairies: immediate post-Senate Bill 27 impact
Source: PeerJ. 2021 Jul 13;9:e11596. doi: 10.7717/peerj.11596 (PMC8284309; doi:10.7717/peerj.11596)

Random Forest Model

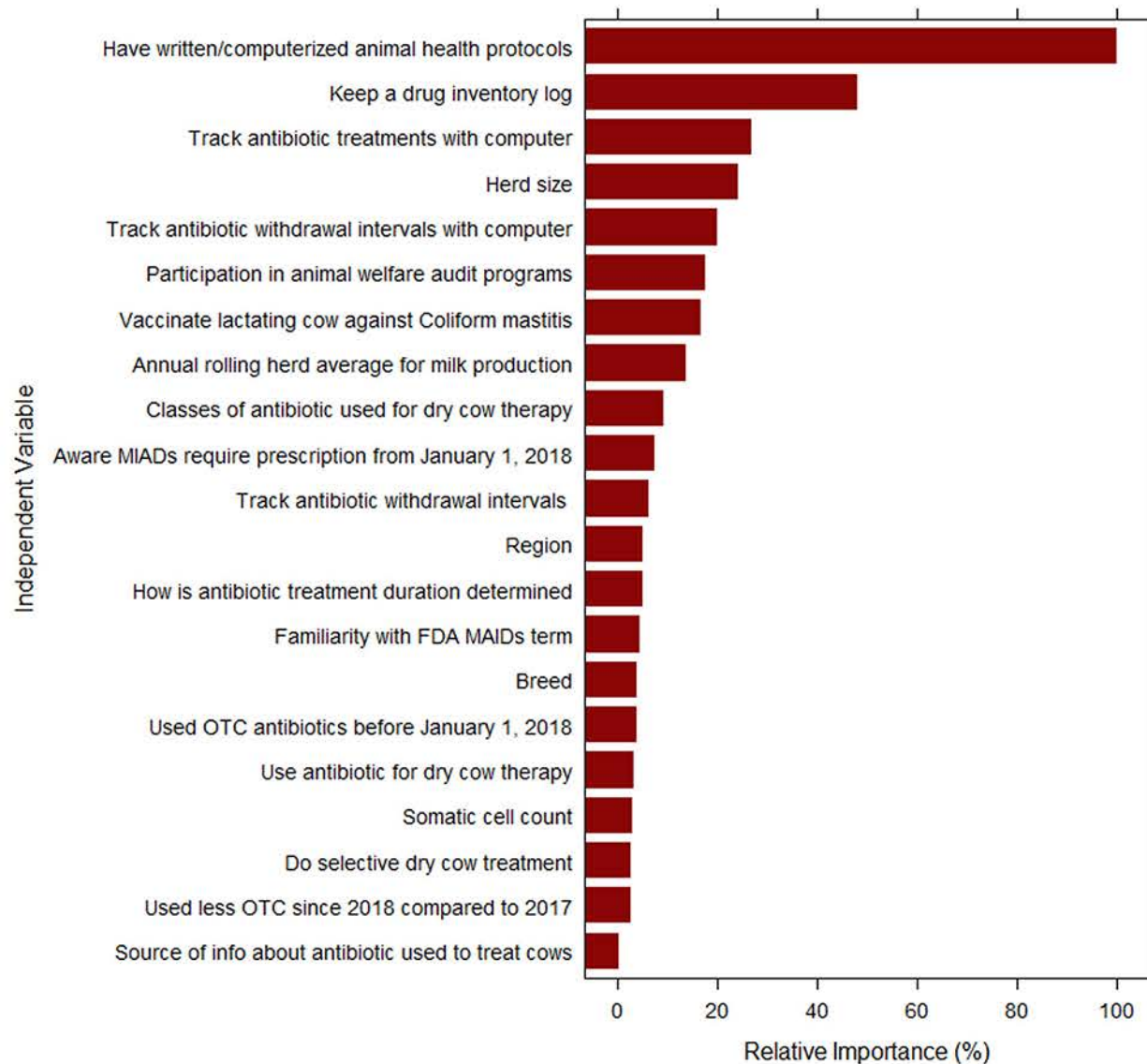

Gradient Boosting Model

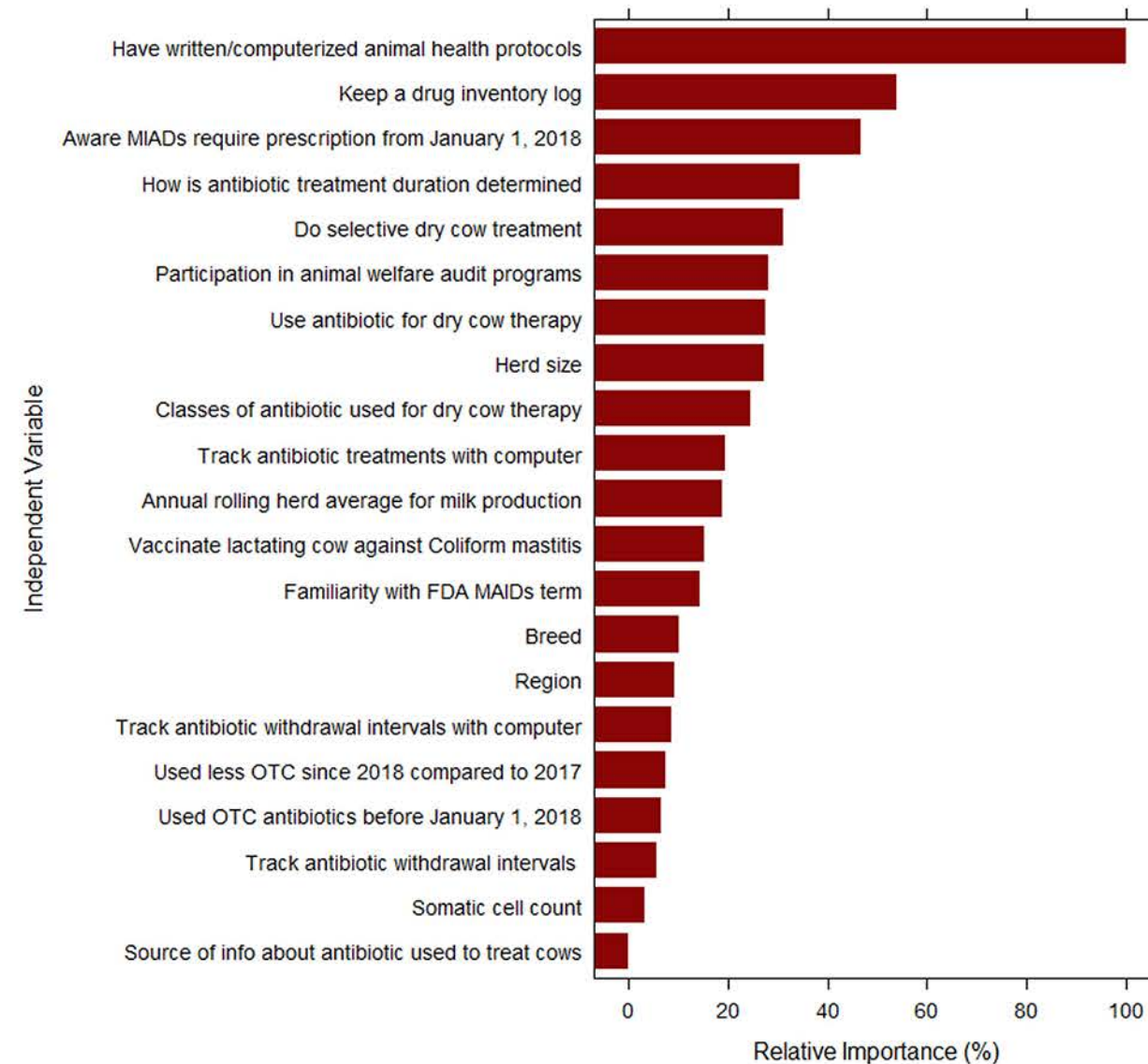

Decision Tree Model

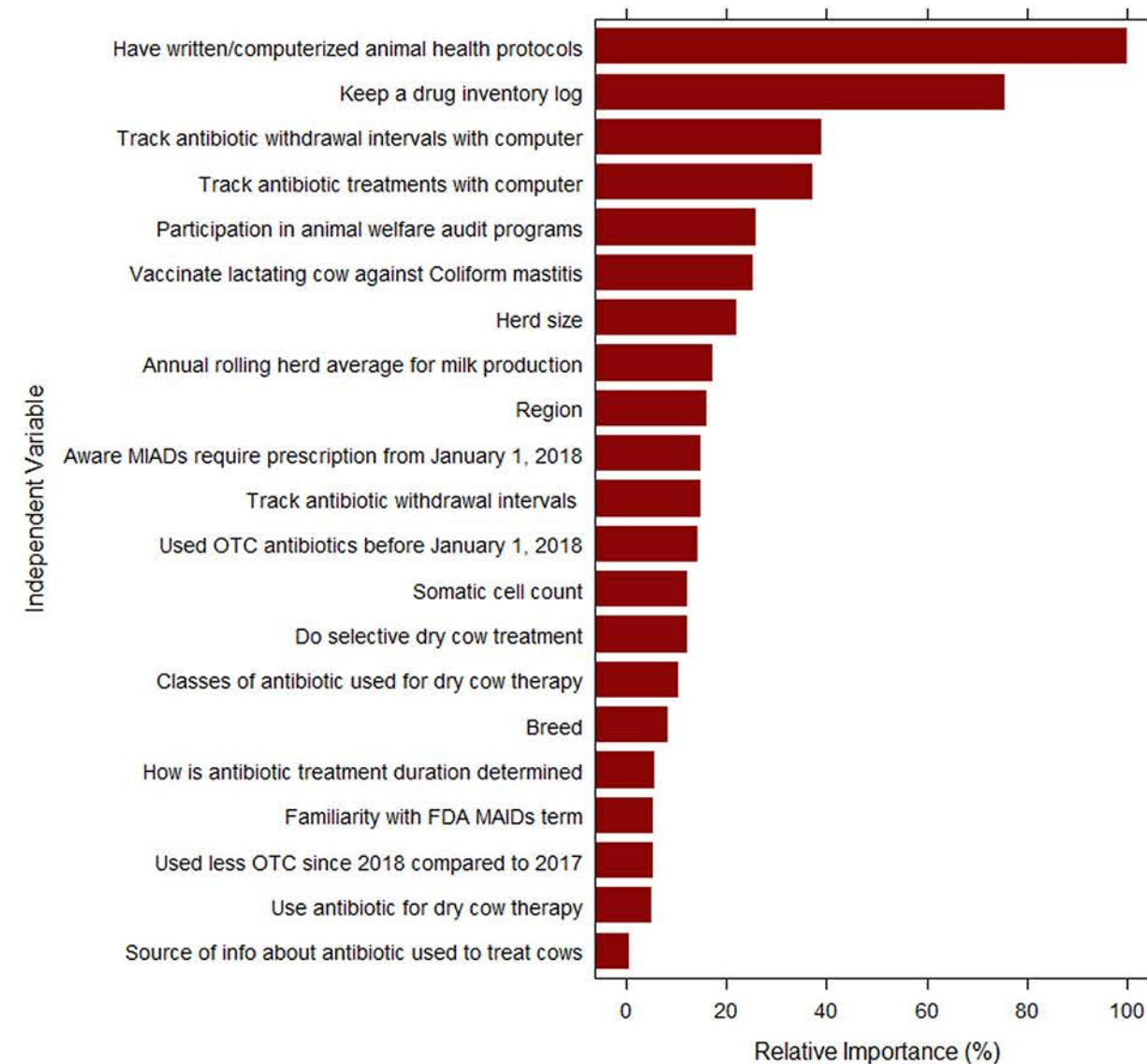

Supplement: Supplemental Information 1 — The rankings were based on three classification algorithms (Decision tree, Random forest, and Gradient boosting) [file peerj-09-11596-s001.pdf]
