# Supplementary material for: 2018 Survey of factors associated with antimicrobial drug use and stewardship practices in adult cows on conventional California dairies: immediate post-Senate Bill 27 impact
Source: PeerJ. 2021 Jul 13;9:e11596. doi: 10.7717/peerj.11596 (PMC8284309; doi:10.7717/peerj.11596)
Supplement: Supplemental Information 2 [file peerj-09-11596-s002.pdf]

Date: ……/………/………   Confidential #:

Survey of Antibiotic Drug Use in Cows on CA Dairies

UC Davis School of Veterinary Medicine & UC Cooperative Extension

- *If you own/ operate several dairies, please answer the questions based on the dairy to which survey was mailed.*
- *Try to answer all the questions, unless prompted to skip a section.*
- *Please return the completed survey in the self-addressed stamped envelope.*

QUESTIONS IN THIS SURVEY REFER TO THE *CURRENT YEAR OF 2018*

SECTION 1: HERD INFORMATION

1. What is your role/position on this dairy? ***Please check all applicable boxes.***

   ☐ Owner   ☐ Herd manager   ☐ Veterinarian   ☐ Other, ***please specify***:_______________

2. In which county in the state of California is this dairy located? _________________________________

3. Is your dairy certified as a producer of organic milk based on USDA standards?

   ☐ Yes   ☐ No

4. What is your herd's average number of milking cows? ___________________________

5. What is your annual rolling herd average (RHA) for milk production? ______________ lbs/cow

6. What was your previous month's average bulk tank somatic cell count (SCC) [cells/ml]? ***Please check only one response.***

   ☐ <100,000   ☐ 100,000 – 199,999   ☐ 200,000 – 299,999

   ☐ 300,000 – 399,999   ☐ 400,000 – 499,999   ☐ >500,000

7. What are the breed(s) in your herd? ***Please check all applicable boxes and fill in approximate percent.***

   ☐ Holstein ______ %   ☐ Crossbred ______ %   ☐ Jersey ______ %   ☐ Other ______ %

SECTION 2: DAIRY COW HEALTH MANAGEMENT & ANTIBIOTIC USE

8. Which of the following dry-off protocols do you use for cows at end of their lactation? ***Please choose only one subsection (a or b)***

   a. ☐ Treat all dry-cows (blanket treatment) with: ***Please check all applicable boxes.***

      ☐ Intramammary dry-cow antibiotics   ☐ Internal teat sealant

      ☐ External teat sealant   ☐ Other, please specify: ______________

   b. ☐ Selective dry cow treatment with: ***Please check all applicable boxes.***

      ☐ Intramammary administration of dry-cow antibiotics based on:

         ☐ SCC   ☐ Mastitis history   ☐ Season/weather   ☐ Milk production

         ☐ Parity   ☐ Culture   ☐ Other, ***please specify:***__________________

      ☐ Internal teat sealant based on:

         ☐ SCC   ☐ Mastitis history   ☐ Season/weather   ☐ Milk production

         ☐ Parity   ☐ Culture   ☐ Other, ***please specify:***___________________

      ☐ External teat sealant based on:

         ☐ SCC   ☐ Mastitis history   ☐ Season/weather   ☐ Milk production

         ☐ Parity   ☐ Culture   ☐ Other, ***please specify:***___________________

9. If antibiotics were selected above in Question 8, which of the following products are used?

☐ Spectramast DC (ceftiofur hydrochloride)

☐ Cefa-Dri®/Tomorrow (cephapirin benzathine)

☐ Boviclox; Dry-Clox®; Dry-Clox® Intramammary Infusion; Orbenin-DC® (cloxacillin benzathine)

☐ Quartermaster® Dry Cow Treatment (penicillin G procaine/dihydrostreptomycin)

☐ Albadry® Plus Suspension (penicillin G procaine/novobiocin)

☐ Other, ***please specify***:____

10. Do you harvest colostrum from fresh cows to feed to newborn calves?

☐ Yes ☐ No

11. Do you have a separate pen, other than the hospital pen, for recently calved cows?

☐ Yes ☐ No

12. Which of the following vaccinations are used in lactating cows? ***Please check all applicable boxes and also specify the approximate lactation stage when vaccines are administered.***

**Disease condition**	**Example vaccine trade names**	**Administration:**		
		**Parity**	**Days In Milk**	**Dry Cow**
☐ Mastitis (coliforms)	Endovac-Bovi, ENVIRACOR J-5, J-5 Bacterin, Mastiguard, J-Vac			
☐ Mastitis (staphylococcus)	Somato-Staph, Lysigin ,STARTVAC			
☐ Diarrhea/ scours (*E. coli*, *Rota*, *Corona* etc.)	ScourGuard 4KC, Bovine Ecolizer, Scour Bos 4			
☐ Respiratory disease	Bovi-Shield Gold, MYCO-B ONE DOSE, TRIANGLE 10 HB, PregGuard, ViraShield, Once PMH IN, Inforce 3			
☐ Abortion and infertility (Leptospirosis, BVD, etc.)	Spirovac VL5, Bovi-Shield Gold, PregGuard, Vibrovax			
☐ Pinkeye	Moraxella Bovoculi Bacterin, Piliguard Pinkeye, 20/20 Vision 7 with SPUR, Pinkeye Shield, Ocu-guard MB-1, I-Site XP			
☐ Clostridium	COVEXIN, Ultrabac 8, Cl. perfringens Type A, Bar Vac			
☐ Footrot	Fusogard			
☐ Other, please specify: ______________________				

13. Currently, which of these sources do you rely on for information about antibiotics used to treat cows? ***Please check all applicable boxes.***

☐ Previous experience with the drug ☐ Other producers ☐ Veterinarian

☐ Product drug label ☐ Magazines/Industry Trade Journals

☐ Drug company material or sales rep. ☐ Local/ National Meetings

☐ Websites (drug co., blogs, etc.) ☐ State/ County/ University Cooperative Extension

☐ FARAD (Food Animal Residue Avoidance Databank) ☐ Other, ***please specify***:____________

14. Who decides which antibiotics are purchased and stocked for treatment of adult cows on your dairy? ***Please check all applicable boxes.***
    a. Antibiotics administered orally (bolus/drench)

       ☐ Owner ☐ Herd manager ☐ Veterinarian

       ☐ Nutritionist (Non-veterinarian) ☐ Other***, please specify:***____________________
    b. Injectable antibiotics

       ☐ Owner ☐ Herd manager ☐ Veterinarian

       ☐ Nutritionist (Non-veterinarian) ☐ Other***, please specify:***____________________

15. Who decides which antibiotic is used to treat a sick cow? ***Please check all applicable boxes.***

    ☐ Owner ☐ Herd manager ☐ Veterinarian ☐ Milker ☐ Treatment crew

    ☐ Nutritionist (Non-veterinarian) ☐ Other***, please specify:***____________________________

16. Do you have written/ computerized animal health protocols (e.g., treatment protocol) for cows?

    ☐ No, ***please SKIP to Question 17.***

    ☐ Yes;
    a. Who developed the protocols? ***Please check all applicable boxes.***

       ☐ Veterinarian ☐ Owner ☐ Herd manager ☐ Nutritionist (Non-veterinarian)

       ☐ Other***, please specify:***____
    b. For which aspects of animal health are these protocols used? ***Please check all applicable boxes.***

       ☐ Disease-specific treatments ☐ Vaccination schedules ☐ Hoof trimming schedule

       ☐ Other***, please specify:***____
    c. *If <u>disease-specific treatments</u> is checked in **b**. above,* which of the following are included? ***Please check all applicable boxes.***

       ☐ Drug dose (by weight, age, drug volume) ☐ Duration (# of times, days of treatment)

       ☐ Milk withdrawal interval ☐ Meat withdrawal interval

       ☐ Not sure about protocol details ☐ Other, ***please specify***:_______________
    d. Who has access to these protocols? ***Please check all applicable boxes.***

       ☐ Owner ☐ Veterinarian ☐ Office Staff ☐ Herd Manager

       ☐ Treatment crew ☐ Nutritionist (Non-veterinarian)

       ☐ I don't know ☐ Other***, please specify:***__________________________________
    e. Are the treatment crew members or milkers trained on treatment protocols for sick cows?

       ☐ No

       ☐ Yes; If yes, who does the training? ***Please check all applicable boxes.***

          ☐ Veterinarian ☐ Owner ☐ Herd Manager ☐ Nutritionist (Non-veterinarian)

          ☐ I don't know ☐ Other***, please specify:***________________________________
    f. How often are these protocols reviewed or revised? ***Please check only <u>one</u> response.***

       ☐ Once to twice a year ☐ Every few years ☐ When a new product is added

       ☐ I don't know ☐ Other, ***please specify:***____________________________________
    g. Who reviews/ revises the protocol? ***Please check all applicable boxes.***

       ☐ Veterinarian ☐ Owner ☐ Herd manager ☐ Nutritionist (Non-veterinarian)

       ☐ I don't know ☐ Other, ***please specify***:____________________________________

17. Do you keep a drug inventory log for your dairy? ☐ Yes ☐ No

18. Which of the following drug-related information do you record? ***Please check all applicable boxes.***

☐ Name of drug ☐ Quantity on hand ☐ Date of purchase ☐ Manufacturer ☐ Drug Supplier/Source
☐ Cost of drug ☐ Drug expiration date ☐ None ☐ Other***, please specify***:____________________

19. How important are the following antibiotic uses / indications on this farm? ***Please check only one box for each row.***

**Antibiotic use/ Indication**	**Very important**	**Important**	**Moderately important**	**Of little importance**	**Not important**
Treat sick animals	☐	☐	☐	☐	☐
Control spread of ongoing disease	☐	☐	☐	☐	☐
Prevent disease in high risk cows	☐	☐	☐	☐	☐

20. How are antibiotic doses for cows usually estimated? ***Please check all applicable boxes.***

☐ Estimate animal weight and use manufacturer's labelled dosage
☐ Estimate animal weight and use a different dosage than the manufacturer's label based on experience or previous treatment outcomes
☐ Estimate animal weight and use the dosage prescribed by veterinarian
☐ Use a standard dose by category of animal, such as first lactation, second or greater
☐ Based on how sick the animal appears
☐ Based on the disease the animal has
☐ Other, ***please specify:***____________________

21. How is the treatment duration (e.g., number of days or number of treatments) determined for cows treated with antibiotics? ***Please check all applicable boxes.***

☐ Follow manufacturer's labelled treatment duration instructions
☐ Follow veterinarian's prescription label treatment duration instructions
☐ Stop the use earlier if animals seem to be recovered (no more clinical signs)
☐ Extend the use if animals still seem to be sick (still have clinical signs)
☐ Based on previous results using the drug on the farm
☐ Other, ***please specify:***____________________

22. Which factors influence selection of a second antibiotic drug to treat a sick animal if the first treatment was not satisfactory? ***Please check all applicable boxes.***

☐ Based on bacterial culture and antibiotic sensitivity results from a laboratory
☐ Based on recommendation from veterinarian
☐ Follow information outlined in the farm's protocol for that disease or condition
☐ Based on previous results using the drug on the farm
☐ Other***, please specify***:____________________

23. Which antibiotic treatment information do you track or record? ***Please check all applicable boxes.***

☐ Date of treatment ☐ Dose ☐ Route
☐ Meat withdrawal interval ☐ Milk withdrawal interval
☐ None ☐ Other***, please specify:***____________________

24. How do you track antibiotic treatments given or administered to cows on your dairy? ***Please check all applicable boxes.***

☐ Computer software, ***please specify:***____

☐ Paper records kept in barn or office ☐ Markings on the animal (e.g., chalk)

☐ White/ chalk board or other temporary marking ☐ Memory

☐ Other***, please specify:***____

25. Do you keep track of antibiotic withdrawal intervals (withholding periods) for treated cows?

☐ No

☐ Yes. ***If Yes, please check all applicable boxes:***

☐ Paper records ☐ Memory ☐ Markings on the animal ☐ White/ chalk board record

☐ Computer software, ***please specify:***__________ ☐ Other***, please specify:***___________

26. Have you submitted any non-routine samples (e.g. milk culture, placenta, cow for necropsy) to a diagnostic lab for diagnosis of infectious diseases in 2018?

☐ Yes ☐ No ☐ I don't know

27. Have any other on-farm diagnostic techniques or procedures such as culture, auscultation (listening to a cow's chest with a stethoscope), lung ultrasound, etc. been used to guide treatment decision with antibiotics for cows?

☐ Yes ☐ No ☐ I don't know

28. Please complete the table below with regard to antibiotics used to individually treat dairy cattle mastitis and metritis on your dairy **since January 1st, 2018. *If you don't use antibiotics, please SKIP to Question 30.***

**Disease condition**	**Average # of cows treated/month**	**Basis for treatment decision** *Please check all applicable boxes.*	**Treatment** *Please check all applicable boxes.*	**Drug used**
**Mastitis**		☐ Rely on findings of abnormal milk<br>☐ California Mastitis Test<br>☐ Milk culture<br>☐ Treat while culture is pending then modify treatment if needed<br>☐ Other, ***specify:***__________	☐ Intramammary antibiotic infusion<br><br>☐ Bolus or injectable antibiotic	1st choice: ________<br>2nd choice: ________<br><br>1st choice: ________<br>2nd choice: ________
**Metritis**		☐ Twins or difficult calving<br>☐ Retained placenta<br>☐ Rely on palpation<br>☐ Vaginal discharge characteristics<br>☐ Take rectal temperature<br>☐ Treat all fresh cows<br>☐ Other, ***specify:***__________	☐ Intrauterine antibiotic<br><br>☐ Bolus or injectable antibiotic	1st choice: ________<br>2nd choice: ________<br><br>1st choice: ________<br>2nd choice: ________

29. Please complete the table below with regard to antibiotics used to treat dairy cattle lameness, pneumonia and postoperative care on your dairy **since January 1st, 2018.** ***If you don't use antibiotics, please SKIP to Question 30.***

**Disease condition**	**Average # of cows treated per month**	**Basis for treatment decision** *Please check all applicable boxes.*	**Treatment** *Please check all applicable boxes.*	**Drug used**
**Lameness**		☐ Rely on lameness signs<br>☐ Hoof trimmer exam<br>☐ Other, ***specify:*** ____________	☐ Hoof treatment (Antibiotic foot wrap, heel spray or foot bath)<br><br>☐ Bolus or injectable treatment	1st choice: ________<br>2nd choice: _______<br><br>1st choice: ________<br>2nd choice: _______
**Pneumonia**		☐ Rely on respiratory clinical signs (cough, difficult breathing, nasal discharge, etc.)<br>☐ Other, ***specify:*** ____________	☐ Bolus or injectable treatment	1st choice: ________<br>2nd choice: _______
**Post-surgery**		☐ Routinely after DA or C-Section<br>☐ Rely on veterinarian instructions<br>☐ Other***, specify:*** ____________	☐ Bolus or injectable treatment	1st choice: _________<br>2nd choice: _______

30. Do you have a veterinarian-client-patient relationship (VCPR) for this dairy?

In California, a VCPR is established when the client has authorized the licensed veterinarian to assume responsibility for making medical judgements and the need for medical treatment of the patient (including the prescription of antimicrobials) AND the veterinarian has assumed that responsibility and has communicated with the client an appropriate course of treatment. For a valid VCPR, the veterinarian must be personally acquainted with the care of the animal(s) by hands-on examination of the animal or by medically appropriate and timely visits to the premises where the animals are kept AND have enough knowledge of the animal(s) to give at least a general or preliminary diagnosis of the medical condition. CCR § 2032.1

☐ No, ***please SKIP to Question 32.***

☐ Yes. → If Yes, which choice best describes your veterinarian? ***Please check only one response.***

☐ Local veterinarian/ clinic   ☐ A technical services veterinarian

☐ Consultant veterinarian   ☐ Other, ***please specify:***___________________________

31. Your VCPR can best be described as: ***Please check only one response.***

☐ A written agreement signed by you and your veterinarian

☐ A verbal agreement between you and your veterinarian

☐ A VCPR was not formally discussed, but I consider that I have one based on the veterinary care my cows receive through my veterinarian

☐ Other, ***please specify:***____

32. How often does your veterinarian observe, monitor, or discuss with you the health of your cows?

☐ Regular intervals, every _______ days/weeks/months. ***Please circle the applicable interval.***

☐ As needed   ☐ Other, ***please specify:*** ____

SECTION 3: PRACTICES AND PERSPECTIVES

33. Do you participate in any of the following animal welfare audit programs for dairy farms? ***Please check all applicable boxes.***

    ☐ The National Dairy FARM Program (**F**armers **A**ssuring **R**esponsible **M**anagement)

    ☐ Validus Dairy Animal Welfare Review Certification ☐ Certified Humane® Program

    ☐ Other, ***please specify***:____

    ☐ None

34. Did you or someone representing your operation receive training or participate in any dairy quality assurance programs in the last 1 year?

    ☐ No ☐ Yes; ***Please specify:***____

35. How familiar are you with the Food and Drug Administration's (FDA) term "medically important antimicrobial or antibiotic drugs"? ***Please check only one response.***

    ☐ Not familiar with medically important antibiotic drugs

    ☐ Heard of medically important antibiotic drugs but not sure how this relates to my dairy

    ☐ I recognize medically important antibiotic drugs are further classified as important, highly important or critically important drugs

    ☐ I recognize that medically important antibiotic drugs are available for livestock only via prescription or veterinary feed directive (VFD) pursuant to a VCPR with a licensed veterinarian

36. Are you aware that since January 1, 2018, all uses of medically important antibiotics in livestock, including injectable antibiotics such as Penicillin Injectable, Liquamycin® LA 200 (oxytetracycline), and Tylan® Injection (tylosin), and boluses, such as Supra Sulfa® III or Sustain III (sulfamethazine), require a VFD or prescription and are no longer sold over-the-counter (OTC) in California?

    ☐ Yes ☐ No

37. **Before January 1, 2018**, which of the following best describes the use of over-the counter (OTC) and prescription antibiotics on this dairy? ***Please check only one response.***

    ☐ Cows were ***not*** treated with OTC antibiotics prior to January 1, 2018.

    ☐ Cows were ***not*** treated with prescription antibiotics prior to January 1, 2018.

    ☐ Cows ***were*** only treated with OTC antibiotics prior to January 1, 2018

    ☐ Cows ***were*** only treated with prescription antibiotics prior to January 1, 2018

    ☐ **Both** OTC and prescription antibiotics were used to treat cows prior to January 1, 2018

38. Since January 2018, what changes did this farm make compared to 2017 with regard to injectable and/or intramammary antibiotics that were previously available OTC? ***Please check all applicable boxes.***

    ☐ Same antibiotics are being used but the dosage or duration **increased**

    ☐ Same antibiotics are being used but the dosage or duration **decreased**

    ☐ One or more antibiotics have been discontinued. ☐ One or more antibiotics have been added.

    ☐ I treat fewer animals with antibiotics ☐ I treat more animals with antibiotics

    ☐ No changes have been made ☐ Other, ***please specify:*** ______________________________

39. Since January 2018, has this farm begun using or increased its use of alternatives to antibiotics?

    ☐ No

    ☐ Yes; I have begun using or increased the use of: ***Please check all applicable boxes.***

    ☐ Vitamins ☐ Minerals ☐ Herbal remedies ☐ Vaccines

    ☐ Other***, please specify:*** ____

40. Since January 2018, have you made changes in management to prevent disease outbreak/ spread?

☐ No

☐ Yes; I have made the following **changes since January 2018**: ***Please check all applicable boxes.***

☐ Made changes or improvements in vaccination programs to prevent disease

☐ Quarantine purchased/returning animals from offsite locations (e.g., fairs, shows, calf ranch, etc.)

☐ Improved biosecurity (e.g., restricted traffic on operation, better isolation of sick animals, or designated separate equipment for feed and manure handling)

☐ Pre-purchase testing of animals before adding to the herd

☐ Other***, please specify:***____

41. Since January 2018, how would you describe:

a. this farm's antibiotic drug costs as compared to 2017 and earlier? ***Please check only one response***

☐ Increased ☐ Decreased ☐ No change

b. animal health on this farm as compared to 2017 and earlier? ***Please check only one response***

☐ Better ☐ Worse ☐ No change

42. Below is an alphabetical list of antibiotic drug use stewardship practices. ***Indicate how important you consider each to be. Please check only one response per row.***

Antibiotic drug use stewardship practice	***Very important***	***Somewhat important***	***Not important***
Administration of appropriate antibiotic drug, dose, route and duration	O	O	O
Good record keeping on treatments and treatment dates	O	O	O
Having a current veterinarian-client-patient relationship (VCPR)	O	O	O
Observing withdrawal periods and drug residue avoidance	O	O	O
Using alternatives to antibiotic drugs (e.g. vaccines, supplements)	O	O	O

43. What is your level of agreement on the following sentences relating to antibiotic resistance? Please check only one response per row.

	Strongly agree	***Agree***	***Neutral***	***Disagree***	***Strongly disagree***
Current antibiotic use practices in animal agriculture will make it harder to treat future livestock infections.	O	O	O	O	O
Antibiotic use in livestock does not cause problems in humans.	O	O	O	O	O
Antibiotic use in livestock leads to bacterial infections in people that are more difficult to treat.	O	O	O	O	O
Any use of antibiotics may result in infections that are more difficult to treat in the future.	O	O	O	O	O
I would be willing to treat my animals with alternatives to antibiotics if they were equally effective.	O	O	O	O	O

You have reached the end of the questionnaire. If you have any additional comments about antibiotic use in dairy cattle please share them in the space below.

Thank you so much for your cooperation.

____

____

____

____
